# Sex-specific genetics underlie increased chronic pain risk in women: genome-wide association studies from the UK Biobank

**DOI:** 10.1016/j.bja.2025.04.013

**Published:** 2025-05-22

**Authors:** Marc Parisien, Matthew Fillingim, Christophe Tanguay-Sabourin, Mathieu Roy, Etienne Vachon-Presseau, Luda Diatchenko

**Affiliations:** 1 Alan Edwards Centre for Research on Pain, McGill University, Montreal, QC, Canada; 2 Department of Anesthesia, Faculty of Medicine and Health Sciences, McGill University, Montreal, QC, Canada; 3 Faculty of Dental Medicine and Oral Health Sciences, McGill University, Montreal, QC, Canada; 4 Faculty of Medicine, Université de Montréal, Montreal, QC, Canada; 5 Department of Psychology, Faculty of Medicine and Health Sciences, McGill University, Montreal, QC, Canada

**Keywords:** chronic pain, genetics, heritability, imaging-derived brain phenotypes, polygenicity, sex differences

## Abstract

**Background:**

Chronic pain disproportionately affects women, but the reasons for this disparity are unclear.

**Methods:**

We investigated this disparity from a genetic perspective using data from the UK Biobank, focusing on multisite chronic pain, which is highly heritable and manifests a sex bias.

**Results:**

Genome-wide association studies (GWAS) revealed that women have approximately 4500 sex-specific causal loci for overlapping pains, four times more than men, accounting for their higher heritability. Heritability partitioning indicated that pain-related loci are primarily enriched in specific brain regions, but only in women. Additionally, 200 imaging-derived brain phenotypes were significantly associated with pain in women, compared with only six in men. GWAS of these brain phenotypes showed stronger genetic correlations with pain in women, particularly regarding cortical thickness and striatal volume. When disentangling pleiotropy from causation in genetically correlated pairs of brain- and pain-related traits, we found that the genetics of brain phenotypes are more often causally implicated with the presence of chronic pain in women.

**Conclusions:**

Our findings suggest that genetics play a crucial role in the increased risk of chronic pain observed in women.

**Editor's key points**

• In a study based on the UK biobank, the authors aimed to determine why and how women experience more chronic pain than men.
• They used multimodal approach combining genetics, patient-reported outcome measures and brain imaging from the large UK Biobank.
• The results show that greater heritability in women is most likely driven by higher polygenicity and that the genetics of brain phenotypes are more often implicated in chronic pain in women compared to men.
• These data constitute an important step towards a genetic explanation as to how and why women experience more chronic pain than men.

One in three patients diagnosed with a chronic pain condition exhibits symptoms of another condition.^1^^,^^2^ Such chronic pain conditions that often co-occur include headaches, back pain, irritable bowel and chronic fatigue syndromes, vulvodynia, fibromyalgia, and temporomandibular disorders, to name a few. When occurring together, these conditions are known as chronic overlapping pain conditions (COPCs).^3^ Central sensitisation resulting from changes in the properties of neurones in the CNS is hypothesised to contribute to the chronification and spreading of pain at multiple body sites, hence the experience of multi-site chronic pain (MSCP).^4^^,^^5^ As such, the exact body location where pain is experienced can become less relevant as the phenomenon leads to a generalised increase in pain sensitivity across the CNS. Furthermore, the presence of multiple and overlapping pain conditions was found to be associated with higher psychological distress and genetic heritability.^6^^,^^7^

The overrepresentation of women among chronic pain patients is well documented,^8^^,^^9^ with women reporting more pain,^10^ displaying lower tolerance for it,^11^ and being more affected by COPC.^12^ The causes for these observations are multi-factorial.^13^ For instance, genetic^14^^,^^15^ and psychosocial factors,^16^^,^^17^ and gender norms and role expectations^18^ have been proposed to explain sex differences with regards to pain. Nonetheless, the National Institutes of Health recently adopted a ‘Sex as a Biological Variable’ policy, emphasising that sex differences might be masked by the usage of males only in preclinical research.^19^^,^^20^ Indeed, the analysis of sex differences in the context of pain research started providing insights into sex-specific molecular pathways of pain.^21^, ^22^, ^23^

Evidence showing reliable sex differences in the CNS contributing to a higher prevalence of chronic pain conditions in women is, however, lacking. As pain is perceived in the brain, noninvasive brain imaging techniques are ideal for probing the structure and function of the brain *in vivo*, enabling the study of the relationships between multimodal brain images and pain.^24^ Both pain phenotypes^25^, ^26^, ^27^, ^28^ and structural and functional brain characteristics^29^^,^^30^ are highly heritable, as evidenced by genome-wide association studies (GWAS). Furthermore, chronic but not acute back pain has been found to have significant heritability in patients with low back pain,^28^ with results likely transposable to chronic pain at other body sites.^31^ The search for genetic underpinnings in chronic pain-relevant tissues often pointed to the CNS rather than the peripheral nervous system.^28^^,^^32^^,^^33^ The scarcity of evidence from previous genetic studies on the subject of sex differences in pain yielded only a few biological insights,^14^^,^^15^^,^^34^ despite the well-documented differences in presentation and aetiology of pain traits in men and women (or males *vs* females animal models).^35^, ^36^, ^37^ Thus, the subject deserves more attention.

In this work, we tackled the basic questions as to why and how women experience more chronic pain than men. To address these questions, we used genetics, self-reported chronic pain, and brain imaging data from the large cohort of the UK Biobank.

## Methods

### Ethics statement

Ethical approval for the UK Biobank was granted by the National Information Governance Board for Health and Social Care and North West Multicentre Research Ethics Committee (11/NW/0382). This study was conducted under UK Biobank's application number 20802. All data provided to us were anonymised.

### Chronic pain

Information regarding chronic pain was derived from the large cross-sectional survey of the UK Biobank project (hereafter UKB).^38^, ^39^, ^40^ Specifically, at each of the eight available body sites X, participants were asked ‘Have you had X pains for more than 3 months?‘. Participants who answered ‘yes’ were deemed patients for chronic pain at site X. The body sites were: (1) head, the site of headaches (UKB field 3799); (2) face (field 4067); (3) neck/shoulder (field 3404); (4) stomach/abdominal (field 3741); (5) back (field 3571); (6) hip (field 3414); (7) knee (field 37773); and (8) widespread (field 2956). Control subjects for all binary traits were those who answered ‘none of the above’ to the question in field 6159 ‘In the last month have you experienced any of the following that interfered with your usual activities? (You can select more than one answer)’. We considered the chronic pain phenotypes collected at the imaging visit (instance 2, 2014+) for those who participated in the brain imaging data collection (∼48 000 people), and for others, the answers were at the initial assessment visit (instance 0, 2006–2010).

### Body site-agnostic chronic pain models

Complimentary to the eight body sites, five additional chronic pain models were defined: (1) the Presence of a Site with Chronic Pain (PSCP) as a binary trait (N_sites_≥1); (2) the Single-Site Chronic Pain (SSCP) as a binary trait (N_sites_=1)^6^; (3) the Multi-Site Chronic Pain (MSCP) as a binary trait (N_sites_≥2)^3^^,^^6^^,^^41^; (4) the Adjacent-Site Chronic Pain (ASCP) as a binary trait, a subset of MSCP; and (5) the Quantitative Multisite Chronic Pain (QMCP), as a quantitative trait (N_sites_).^32^^,^^42^ Case subjects for SSCP were those who answered ‘yes’ to any *one* of the seven body sites defined above (excluding widespread pain). Case subjects for MSCP were those who answered ‘yes’ to *two or more* of the seven specific body regions, including those who answered ‘yes’ to widespread pain. Case subjects for PSCP were those from SSCP plus those from MSCP. Case subjects for ASCP were those who answered ‘yes’ to at least one pair of adjacent chronic body sites, excluding widespread pain. Adjacent body sites were: head + face, face + neck/shoulder, neck/shoulder + stomach/abdominal, stomach/abdominal + back, back + hip, and hip + knee. Counts of chronic pain sites for an individual in QMCP were the number of ‘yes’ answers, ranging from 0 to 7 (excluding widespread pain), as previously defined.^32^

### Imaging-derived brain phenotypes

Imaging-derived brain phenotypes (IDBPs) were sourced from the UK Biobank, encompassing a comprehensive range of structural and functional brain metrics. These IDBPs were extracted using three primary neuroimaging techniques: T1-weighted MRI, diffusion MRI (dMRI), and resting-state functional MRI (fMRI). The methodology for image processing included steps for artifact elimination, ensuring consistency across modalities and participants through standardised image alignment, rigorous quality control measures, and the subsequent derivation of phenotypes. Detailed descriptions of these procedures are available in the UK Biobank's central brain imaging documentation (https://biobank.ctsu.ox.ac.uk/showcase/showcase/docs/brain_mri.pdf) and have been elaborated upon by Alfaro-Almagro and colleagues.^43^ Specifically, the imaging data were curated and made available by the UK Biobank as part of Category 508 (‘UKB brain imaging pipeline’), including field 25753 (‘rfMRI partial correlation matrix, dimension 100’) under Category 111 (‘resting functional brain MRI’).

The T1-weighted MRI data encompassed 1041 features, including cortical thickness, surface area, volume, and regional grey/white matter intensity contrast, and also volumes of regional and subcortical grey matter (refer to the pdf document above for more details). For the dMRI modality, data from 39 100 participants were assessed, focusing on 614 distinct features. These features encompass microstructural characteristics of white matter tracts, such as mean fractional anisotropy and mean diffusivity, along with diffusion tensor imaging derivatives such as orbital diffusivity, mode of anisotropy (MO), axial diffusivity (L1), radial diffusivity (L2), intracellular volume fraction (ICVF), and isotropic volume fraction (ISOVF).

The resting-state fMRI data included 210 features. These data were processed using the minimally preprocessed pipeline developed by the FMRIB group at Oxford University, UK. Key preprocessing steps applied to the UK Biobank's resting-state fMRI data included motion correction via MCFLIRT, grand-mean intensity normalisation, high-pass temporal filtering, fieldmap unwarping, and gradient distortion correction. Additionally, noise components were identified and mitigated using FSL ICA-FIX. Resting-state networks were generated by applying temporal processing and variance normalisation followed by advanced group-principal component analysis (PCA) and independent component analysis (ICA) techniques to identify distinct brain components. This process generated spatial maps representing brain networks. Non-neuronal components were removed, and the remaining data were used to create individual partial correlation-derived connectivity matrices, which were used in this study. Comprehensive details on the preprocessing methods used by the UK Biobank are documented in the published literature.

### Genome-wide association studies

All GWAS were run using regenie version 2.2.4^44^; binary traits ran using the programme option ‘—bt’. The advantages of using regenie were: (1) it dealt with cryptic relatedness; (2) it dealt with case/control imbalances; and (3) it ran fast for quantitative traits as it can fully utilise computers with hundreds of cores. Chronic pain covariables were: age, age squared, genotyping array, the first 40 genetic principal components, and dummy-coded recruitment sites. Sex was defined as the canonical, genetically ascertained sex (XY = man, XX = woman; field 22001). Sex was not considered as a covariable for sex-stratified analyses. IDBP covariates included all those used in chronic pain analyses, plus: mean head motion during the resting-state fMRI scan (Motion_T2, UKB field 24438; MotionSquared; MotionCubed), head size scaling parameters (HeadScaling_T2, field 25000; HeadScalingSquared_T2; HeadScalingCubed_T2), scanner position coordinates (ScannerXPosition_T2, field 25756; ScannerYPosition_T2, field 25757; ScannerZPosition_T2, field 25758; and ScannerPosition_T2, field 25759), and imaging assessment centre (BinnedSite_T2, dummy-coded as multiple binary indicators). These covariables were selected from a detailed analysis of confounders for brain imaging data,^45^ with considerations of orthogonality with pain.^7^ For regenie's step 1, a total of 93 000 single-nucleotide polymorphisms (SNPs) were considered; these markers were retained by the UKB for kinship estimation (column ‘in_Relatedness’ of the ‘Marker-QC’ file). About 1 million tested SNPs in regenie's step 2 were those from the European subset of the pan-UK Biobank project (Pan-UKB team; https://pan.ukbb.broadinstitute.org. 2020). These SNPs are high-quality HapMap 3 variants that are (1) in autosomes, (2) not in the Major Histocompatibility Complex (MHC) region, (3) bi-allelic SNPs, (4) with INFO score >0.9, and (5) minor allele frequency (MAF) >1% in the UKB and in the Genome Aggregation Database (gnomAD) genome/exome (if available). The smallest tolerated Hardy–Weinberg equilibrium *P*-value was set at 10^−12^. Retained individuals were those of European ancestry (‘Caucasians’; field 22006), whereas those who displayed failed genotyping quality controls, peculiar heterozygosity, sex chromosomes aneuploidy, or opted out of the study at the time of the start of this study (circa December 2021) were excluded (sample-QC file from resource 531).

### Post-GWAS processing

Genomic inflation factor, confounding bias ratio, and total narrow-sense heritability computed with the LD Score regression (LDSC) program.^46^ Genomic inflation factors (λ) showed an elevated departure from the ideal value of 1, more so for PSCP, QMCP, ASCP, and MSCP (1.2–1.5) than for SSCP (∼1.1; Supplementary Fig. 1; Supplementary Table 1), but proportional to sample sizes (λ_M+W_ > λ_W_ > λ_M_) as previously observed.^46^ However, LD-corrected genomic inflation factors were all close to 1, indicating limited inflation of test statistics from artifacts of population stratification or other confounding factors.^46^ Genomic inflation factor ratios indicated that at most 20% of inflation could be attributed to factors other than genetics. Number of causal variants, discoverability, and polygenicity estimates were obtained by univariate analyses, and shared number of causal variants between MSCP and IDBPs by bivariate analyses using MiXeR.^47^^,^^48^ Tissue-based partitioned heritability was estimated using LDSC,^49^^,^^50^ on genes specifically expressed in hundreds of tissues and cell types by Benita and colleagues.^51^ From the 126 tissues/cell types available, we plotted only 106 of those, removing those classified in ‘airway’, ‘endocrine’, ‘heart’, ‘liver’, ‘testis’, and ‘thyroid’. Genetic and environmental correlations between MSCP and IDBPs were obtained using the GECKO program.^52^ Conversion of observed scale heritability estimates to the liability scale ones has been performed using equation 23 of Lee and colleagues,^53^ assuming that the population prevalence is well reflected in the UKB cohort (the no ascertainment hypothesis).

### Phenotypic association

Logistic regressions were performed between each IDBP and the presence of MSCP. To do so, study participants were matched by age and by the presence of MSCP such that equal numbers of men and women were considered (same number of men and women, and same number of case men and case women). IDBPs were Z-scored such that each IDBP would have a mean of 0 and a standard deviation (sd) of 1. Z-scoring was done in a sex-stratified manner as each sex might display varying means and variances. Covariables were the same as for IDBP GWAS.

### Statistical significance for sex difference

The statistical significance of a difference in point estimates (E) between men (M) and women (W), given their standard errors (se), was estimated using Z = | E_W_ – E_M_ |/√(SE^2^_W_ + SE^2^_M_), where *P*-value was obtained using *P*=2∙pnorm(-| Z |). se from sd were obtained from MiXeR's 20 randomised replicates: se=sd/√(20). The sex bias (Δ) for a given IDBP modality (e.g. ‘thickness’) or a brain region (e.g. ‘striatum’) with N labels was first assessed by plotting sex-stratified cumulative distribution functions, then estimated with the average horizontal ‘displacement’ between the women's (W) and men's (M) distributions across all i^th^ data points; Δ = N^−1^ ∑^N^_i_ | X^W^_i_ – X^M^_i_ |.

### Visualisation of brain maps

Brain colourmaps were rendered with the help of the ENIGMA toolbox.^54^ Plotted values were genetic correlations between IDBPs and MSCP on the absolute scale (|R_g_|), but only for those IDBPs phenotypically associated with MSCP at the false discovery rate (FDR) of 10% level.

### Data availability

Tissue- and cell-based gene expression data by Benita and colleagues^51^ are publicly available at http://xavierlab2.mgh.harvard.edu/EnrichmentProfiler/download.html. All other data supporting the findings of this study are available through application to the UK Biobank or by request to the corresponding author.

## Results

### Generalised models for chronic pain

**Fig 1:**
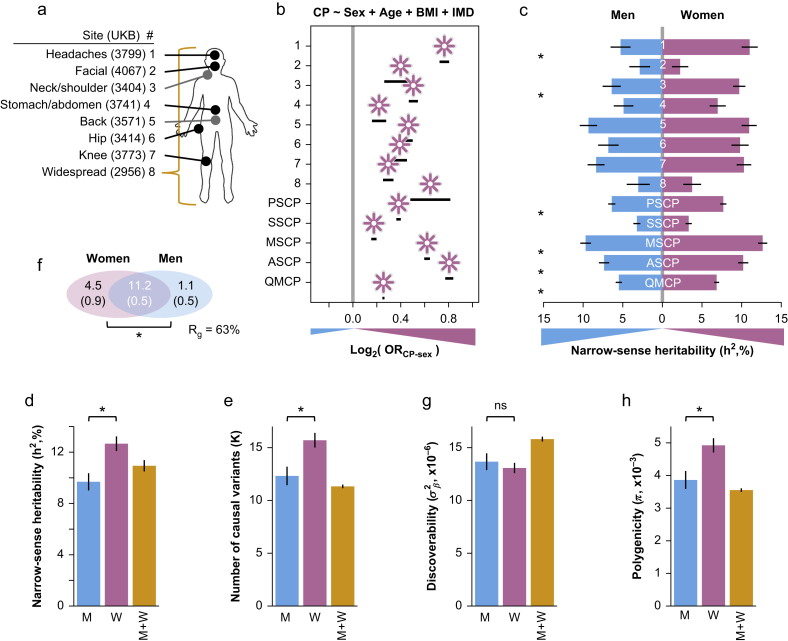
Sex differences in the phenotypic and genetic structures of general models for chronic pain (CP). (a) Body sites in the UK Biobank (UKB) cohort were surveyed for the self-declared presence of CP. Sites are sequentially numbered from 1 to 8, starting at the head. For each body site, the UKB field is shown. (b) Odd ratios between sex and CP. Reported are the odds for the presence of CP with respect to sex, adjusted for age, body mass index (BMI), and index of multiple deprivation (IMD), as potential confounders of CP. Odds estimated for each body site (1–8), and for selected generalised CP models: Presence of a Site with Chronic Pain (PSCP); Single-Site Chronic Pain (SSCP); Multi-Site Chronic Pain (MSCP); Adjacent-Site Chronic Pain (ASCP); and Quantitative Multisite Chronic Pain (QMCP). Stars indicate point estimates and segments indicate their 95% confidence intervals. Positive values indicate increased odds for CP in women (reddish-purple). Shown for QMCP is the exponential of the linear regression's effect as pseudo-odds (log_2_-scaled). (c) Heritability of CP. Narrow-sense heritability is shown for each body site and CP models, tracked separately for men (left, blue) and women (right, reddish-purple). A black segment on each bar indicates standard error. ∗Significant sex difference at the FDR 10% level. (d–h) Sex-specific genetic architectures of MSCP. (d) Narrow-sense heritability, in men plus women combined (M+W, yellow), in men only (M, blue), and women only (W, reddish-purple). (e) Estimated number of causal variants. (f) Venn diagram showing the estimated number of causal variants that are shared (in thousands) and sex-specific. Numbers in parentheses are standard errors of the estimates. ∗Significant difference (*P*<0.05) in the estimated number of sex-specific causal variants. The genetic correlation is also shown (Rg). (g) Discoverability. (h) Polygenicity.

We first conducted a study to determine whether chronic pain was more prevalent in women in the UK Biobank cohort. We used logistic regression to test the association between sex (men, 0; women, 1) and presence of chronic pain (yes, 1; no, 0) while accounting for possible confounding factors such as age, BMI, and index of multiple deprivation.^55^ For all eight individual body sites surveyed (Fig. 1a; Supplementary Table 2a), our findings revealed that women have significantly higher odds of chronic pain (Fig. 1b; Supplementary Table 2b).

We next tested several body site-agnostic models of chronic pain (see Supplementary Methods for model definitions). Sex odds ratios for chronic pain models were also estimated (Fig. 1b; Supplementary Table 2b). All generalised pain models exhibited sex differences in which women displayed increased odds for the presence of chronic pain. The chronic pain data from the UK Biobank thus recapitulated the sex differences observed in other cross-sectional studies of chronic pain.^56^, ^57^, ^58^

We then conducted sex-stratified GWAS for the eight different individual chronic pain body sites and the generalised pain models. From the GWAS summary results, we extracted the narrow-sense heritability (h^2^), the trait's percent variance explained by genetics at common variants, and found that all estimates were significant (FDR <4%; Supplementary Table 2c). We observed that the estimated heritability was significantly higher in women than in men at each body site, except for the facial site (Fig. 1c). SSCP did not exhibit a significant sex difference for heritability, whereas other generalised models did (sex difference FDR <7%). Among the tested models, MSCP best captured the sex bias towards women and was the most heritable.

### Sex differences in the genetic architectures of chronic pain

We next aimed to dissect the sex-differentiated autosomal genetic architectures of human chronic pain, with the main goal of revealing underlying sex-specific differences. We revisited heritability estimates for all chronic pain models (Supplementary Fig. 2a; Supplementary Table 3a). MSCP displayed the largest sex-combined estimates (M+W 11%; *P*<3×10^−13^). Furthermore, women displayed significantly larger sex-stratified heritability estimates than men, with sex-combined values middling between sex-specific values. MSCP heritability was 3% higher in women than in men (12.7% *vs* 9.7%), a considerable 1.3-fold increase (difference *P*=8×10^−4^; Fig. 1d). The largest narrow-sense heritability estimate was for women at about 13% for MSCP (Supplementary Fig. 2a). Assuming the absence of ascertainment, that is, the population prevalence is equal to sample's one (as the UKB was not specifically a case/control study of MSCP but rather a cross-sectional one), the heritability of MSCP on the liability scale was estimated at 20.9% for women (MSCP prevalence in the UKB at 36.4%) and 17.0% in men (29.2% prevalence) (Supplementary Table 2c). We thus reaffirmed MSCP as the most heritable pain model, even more so in women than in men.

The heritability of a trait is proportional to its estimated number of causal variants, defined as those explaining 90% of the heritability of a trait. About 11 300 causal variants were estimated for sex-combined MSCP (Supplementary Fig. 2b; Supplementary Table 3b). Women, however, displayed significantly greater estimates than men for MSCP (difference *P*=3×10^−3^). As such, the number of causal variants in women was about 1.3-fold greater than in men, at 15 700 *vs* 12 300 variants (Fig. 1e). We also calculated the overlap of causal variants shared between men and women (Supplementary Fig. 2c; Supplementary Table 3c). Significant sex-specific differences in causal variants were detected for MSCP (difference *P*=1×10^−3^; Fig. 1f), with four-fold more unique variants in women than in men (4500 *vs* 1100). Furthermore, for MSCP, the number of causal variants unique for women was about 30% of women's total causal variants, whereas that for men was only 9%. Overall, women displayed a significantly larger genetic footprint for chronic pain than men.

Phenotypic covariances were further decomposed into genetic (Rg) and environmental (Re) subcomponents (Supplementary Fig. 2c; Supplementary Table 3d). In MSCP, the environmental correlation between sexes was negligible (∼1%), with a moderate value of intersex genetic correlation (∼63%) (Fig. 1f). Within-trait, between-sex genetic correlations for neuropsychiatric and behavioural traits have been reported much higher (e.g. Fig. 1e of Martin and colleagues^59^ where the majority of tested traits displayed >80% within-trait, between-sex genetic correlation estimates). The moderate between-sex genetic correlation is attributed in part to the vastly different number of sex-specific causal loci for MSCP.

To further understand the genetic architecture of chronic pain and sex differences, we decomposed heritability into its constituent components of polygenicity (the proportion of causally associated SNPs) and discoverability (the average effect size strength of causally associated SNPs). Discoverability estimates for MSCP were the largest among all generalised chronic pain models (Supplementary Fig. 2d; Supplementary Table 3b) but did not show significant sex differences (Fig. 1g). Sex-stratified estimates of polygenicity were significantly different between men and women, including for MSCP (difference *P*=3×10^−3^) (Supplementary Fig. 2e; Supplementary Table 3b). Polygenicity of MSCP was found larger in women than in men by ∼1.3-fold (Fig. 1h). Thus, the observed heritability difference between sexes was owing to the difference in polygenicity, and not because of differences in discoverability. To address the disproportionate statistical power to detect GWAS effects as a result of the larger cohort of women, we replicated these results in a GWAS performed in a cohort assembled from a random subset of women but with matched numbers of cases and controls as in men. These results explain why a difference in the number of causal SNPs was observed between sexes as the number of causal variants is proportional to the polygenicity. Overall, no significant genetic effect size differences were detected between the sexes, but women showed a larger number of causal variants for MSCP than men, alluding to an extended genetic footprint.

### Sex differences in the tissue-based heritability of chronic pain

**Fig 2:**
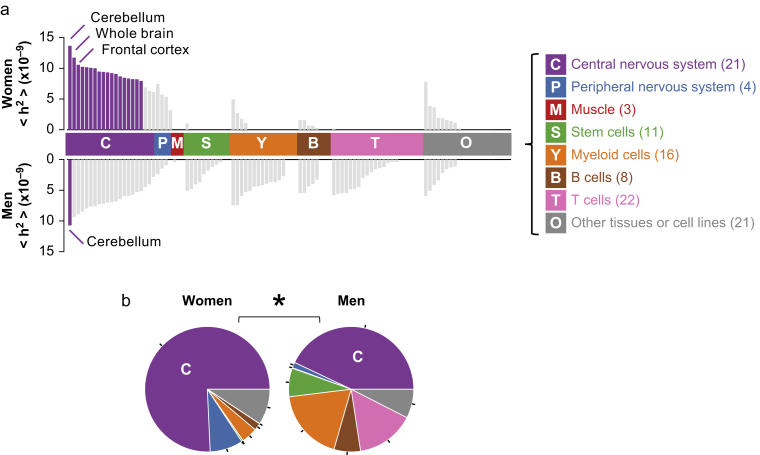
Sex differences in the tissue-based partitioned heritability of muti-site chronic pain. (a) Per-single nucleotide polymorphism average heritability (h^2^) at loci of genes specifically expressed in selected tissues or cell lines. Tissues and cell lines are coloured by broad classes, depicted on the right (number of tissues/cell types per class indicated between parentheses). Insignificant tissues with false discovery rate >10% are coloured light grey. The top plot is for women, and the bottom plot is for men. (b) Relative contributions of tissue classes to partitioned heritability, in women (left) and in men (right). The colour coding of the pies corresponds to panel (a). ∗When *P*<0.05 for the difference in heritability between men and women for the central nervous system class.

We next performed tissue-based partitioned heritability to uncover tissues and cell types for which loci at specifically expressed genes carry excess heritability for chronic pain (Supplementary Fig. 3; Supplementary Tables 4a–j). Partitioning heritability yielded per-SNP average heritability estimates for loci at genes expressed in tissues of interest, denoted as ‘h^2^’. Women displayed significantly enriched heritability in tissues of the CNS compared with men for MSCP (Fig. 2a). Moreover, heritability enrichment in men was more spread out across tissues, albeit almost exclusively in a non-significant manner for any particular one. Stem and immune cell types (myeloid, B, and T) expressed some enrichment signal but only in men (again in a non-significant manner). Only the cerebellum region was found significantly enriched for MSCP in both sexes (Fig. 2a), but women displayed as many as 17 other brain regions significantly enriched after correction for multiple testing (Supplementary Table 4e). Tests for sex differences at the single tissue level did not identify significant differences, although heritability coefficients ‘h^2^’ in the CNS for MSCP trended larger for women than for men (Supplementary Tables 4k–o).

When grouping tissue coefficients in broad classes (Supplementary Tables 4p–t), a significant sex difference in the enrichment of heritability in CNS tissues was detected for MSCP, with most of the enriched heritability signals found in brain regions for women compared with men (pie charts relative heritability >75% in women; ∼40% in men, difference *P*<0.05) (Fig. 2b). The largest class-based enrichment was found in the CNS and accounted for about 85% in women for the ASCP phenotype (Supplementary Table 4s). Overall, partitioned heritability analyses unveiled significantly enriched heritability signals concentrated at loci of genes exclusively expressed in several brain regions in women but not in men for MSCP and other chronic pain models (Supplementary Fig. 3a–e).

### Sex differences in the phenotypic association between brain features and multi-site chronic pain

**Fig 3:**
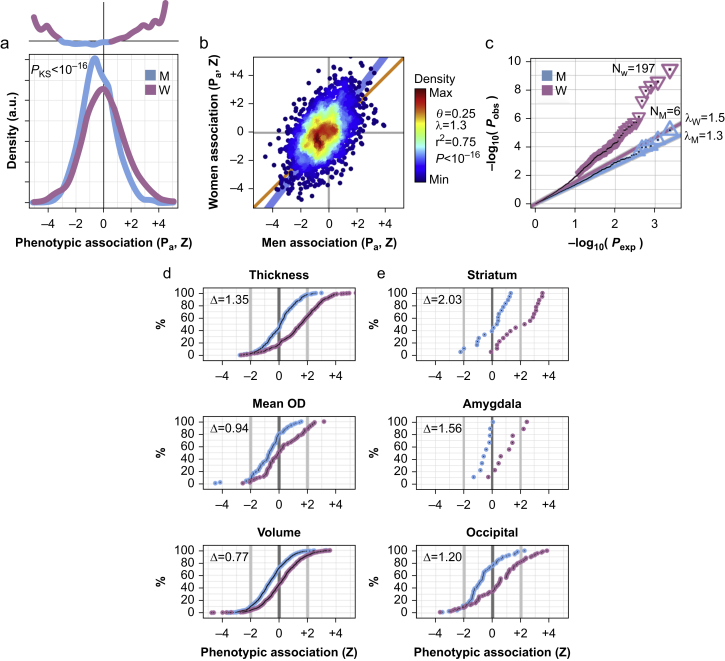
Sex differences in the phenotypic association between imaging-derived brain phenotypes (IDBPs) and multi-site chronic pain (MSCP). (a) Sex-stratified distributions of association test statistics (Z) between IDBPs and MSCP. The difference between men and women is quantified using a two-sided Kolmogorov–Smirnov test, for which the *P*-value is shown (*P*_KS_). In the inset at the top is a curve tracking the log2 ratio of the distributions of Y values in women to men. (b) Correlation of phenotypic association test statistics between men (X-axis) and women (Y-axis). Each dot is an IDBP. Dots are coloured by the density of data points, from minimum (dark blue) to maximum (red). Linear regression intercept (*θ*), slope (*λ*), percent variance explained (*r*^2^), and *P*-value (*P*) are also shown. (c) Sex-stratified QQ plots, showing observed *P*-value (*P*_obs_) for the association between IDBPs and MSCP *vs* that expected (*P*_exp_). Sex-stratified slopes (*λ*) of the QQ plot's data are shown. Significant IDBPs at the false discovery rate of 10% level (N) are highlighted in women using downward-pointing triangles and in men using upward-pointing ones. (d and e) Sex bias of association strengths between IDBPs and MSCP. The bias is highlighted with cumulative distribution plots that track the percentage (%) of IDBPs as a function of association strength test statistics (Z) with MSCP. Each dot is an IDBP, stratified by sex (women, magenta; men, blue). The sex bias (Δ) is the average horizontal distance between women's and men's data points. A thick black vertical line indicates no effect (Z=0), whereas thick grey vertical lines indicate test statistics values of Z=plus or minus 2, with associated nominal raw *P*-values of 0.05. (d) Top three most biased IDBP modalities: thickness, mean orientation dispersion (OD), and volume. (e) Top three most biased brain regions: striatum, amygdala, and occipital.

Having established that women displayed enriched heritability at brain gene loci, we sought to map the phenotypic association between the various IDBPs and the presence of MSCP via logistic regression (Supplementary Table 5). Regressions were performed with matched numbers of cases and controls in each sex-specific cohort. After correction for multiple comparisons, we found no significant sex differences in effect estimates for the association between IDBPs and MSCP on a per-IDBP basis (FDR >59%). However, the sex-stratified distributions of association test statistics significantly differed (two-sided Kolmogorov–Smirnov test *P*-value *P*_KS_<10^−16^). Overall, women displayed stronger test statistics than men, in both positive and negative association directions (Fig. 3a). Importantly, the cases and controls were matched in numbers between men and women for this analysis to avoid the artificial enhancement of the women-specific test statistics owing to larger sample size for women in the UKB cohort. The correlation of test statistics between men and women was strong (percent variance explained 75% *P*<10^−16^; Fig. 3b). However, the correlation's slope of 1.3 suggested that for any given value of the association test statistic observed in men, the corresponding value in women would be as much as 30% higher (as a linear trend). Sex differences were emphasised using QQ plots (Fig. 3c). In men, the observed association *P*-values followed what was expected (albeit a slope *λ* of 1.3), whereas, in women, the observed association *P*-values displayed an upward trend, departing from its own expected values (*λ*=1.5). The QQ plots slopes >1 indicated that association test statistics were inflated for both sexes, reflecting that IDBPs were not independent of one another. Overall, women displayed much stronger associations between IDBPs and MSCP, with 197 significant IDBPs at the FDR 10% level, whereas in men only six significant IDBPs were found (Fig. 3c; Supplementary Table 5).

To understand more about IDBPs that were strongly associated with chronic pain in women, we proceeded with analyses for enrichment of labels via hypergeometric tests. We identified three imaging-derived brain modalities (‘labels') that were significantly associated with MSCP in women within the top 10% of associated IDBPs: 'thickness', representing various cortical thickness phenotypes (FDR <8×10^−16^); ‘area’, representing the surface area of cortical regions (FDR 4%); and ‘partial corr’, representing partial correlation connectomes derived from resting-state functional connectivity (FDR 6%) (Supplementary Table 6a). Significant (FDR 6%) but anecdotal (number of IDBPs ‘q’ less than 10) enrichment values were found in men, but not for ‘thickness’, ‘area’, nor ‘partial corr’ like in women (Supplementary Table 6b). Similarly, in the top 10% most associated brain regions namely the striatum (FDR <1%), parietal (FDR <1%), and occipital (FDR 1%) were enriched for significant associations in women (Supplementary Table 6c) but not in men (Supplementary Table 6d).

We next focused on the sex differences in association strengths for various brain imaging modalities with chronic pain. Among all IDBPs, a total of 306 IDBPs featured the label ‘thickness’, and their association strengths were plotted in a sex-stratified manner (Fig. 3d). Women displayed 40% of the data with association test statistics greater than +2 (equivalent to nominal *P≤*0.05), whereas men displayed only a few percent. To superimpose men's curve on top of women's, we would have to shift men's data rightward by an average of Δ=1.35 units of test statistics, thus indicating that in women, the association of ‘thickness’ with chronic pain was stronger than that for men. Positive association test statistics showed that greater cortical thickness was associated with greater odds for the presence of MSCP. The rightward shift was also observed for ‘mean orientation dispersion’, a measure of white matter tract organisation, and for ‘volume’ labels, which represent cubic volumes of global brain regions (Fig. 3d). A positive displacement was observed for each tested modality (Supplementary Table 6e). A census of brain regions indicated that the striatum, amygdala, and occipital cortex were the top three brain regions that displayed the largest sex biases (Fig. 3e). All brain regions also displayed a rightward shift for greater association test statistics in women than in men (Supplementary Table 6f; IDBPs to brain regions mapped in Supplementary Table 6g). Overall, this indicated that many IDBPs in various brain regions are more strongly associated with chronic pain in women than in men.

### Sex differences in the genetic architectures of brain features

**Fig 4:**
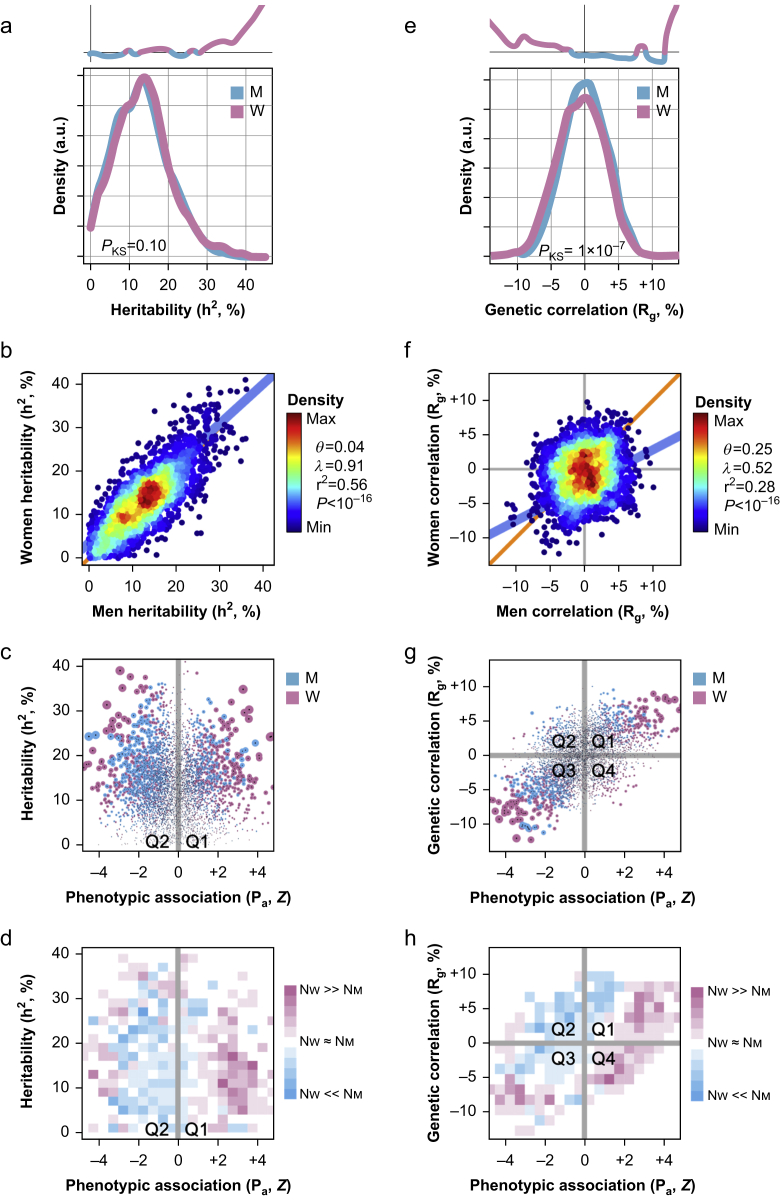
Sex differences in the genetic architecture of imaging-derived brain phenotypes (IDBPs). (a–d) Sex differences in the heritability of IDBPs. (a) Sex-stratified distributions of narrow-sense heritability estimates of IDBPs. The difference between men and women is quantified using a two-sided Kolmogorov–Smirnov test, for which the *P*-value is shown (*P*_KS_). In the inset at the top is a curve tracking the log2 ratio of the distributions of Y values in women to men. (b) Correlation of heritability estimates between men (X-axis) and women (Y-axis). Each dot is an IDBP. Dots are coloured by the density of data points, from minimum (dark blue) to maximum (red). Linear regression intercept (*θ*), slope (*λ*), percent variance explained (*r*^2^), and *P*-value (*P*) are also shown. (c) Scatter plot tracking heritability estimates as a function of phenotypic association with multi-site chronic pain (MSCP). Each dot is an IDBP coloured by sex. (d) Heatmap tracking per-sex counts of IDBPs in each subregion. (e–h) Sex differences in the genetic correlation of IDBPs and MSCP. (e) Sex-stratified distributions of genetic correlation estimates (R_g_) between IDBPs and MSCP. The difference between men and women is quantified using a two-sided Kolmogorov–Smirnov test, for which the *P*-value is shown (*P*_KS_). In the inset at the top is a curve tracking the log2 ratio of the distributions of Y values in women to men. (f) Correlation of R_g_ values between men (X-axis) and women (Y-axis). Each dot is an IDBP. Dots are coloured by the density of data points, from minimum (dark blue) to maximum (red). Linear regression intercept (*θ*), slope (*λ*), percent variance explained (*r*^2^), and *P*-value (*P*) are also shown. (g) Scatter plot tracking R_g_ as a function of phenotypic association between IDBPs and MSCP. Each dot is an IDBP coloured by sex. (h) Heatmap tracking per-sex counts of IDBPs in each subregion.

We next tested if the observed sex differences in phenotypic associations between IDBPs and chronic pain could be attributable, at least in part, to genetics. To do so, we performed sex-stratified GWAS on 2319 IDBPs in the UKB cohort. From the summary of GWAS results, we extracted the heritability estimates (Fig. 4a; Supplementary Table 7a). Sex-stratified distributions of heritability estimates were found to not significantly differ overall (*P*_KS_>0.05), but women featured more IDBPs with larger heritability estimates than men in the 30–45% heritability range. Nonetheless, heritability estimates in women correlated with those in men, with a percent variance explained of 56% (*P*<10^−16^; Fig. 4b).

For our study purposes, we focused on the IDBPs which were strongly phenotypically associated with chronic pain. We thus used a scatter plot that tracked two variables of interest, more specifically the genetic heritability of IDBPs *vs* the phenotypic association of IDBPs with MSCP, a genotype-by-phenotype (G×P) plot (Fig. 4c). There, we qualitatively observed more IDBPs in women that were simultaneously heritable and phenotypically associated with chronic pain. To quantify this enrichment, we partitioned the X and Y axes into 20 equally spaced squared subregions, then counted the number of IDBPs for men and women in each subregion (Fig. 4d). We found that women featured more IDBPs than men for subregions of Z<–3 and Z>+2 at any level of heritability. An enrichment for women was also found in another genetic parameter concerned with the adaptation to evolutionary pressure that can be estimated using the evolutionary response to selection, so-called evolvability.^60^ Evolvability is distinct from and uncorrelated with heritability.^61^ A rigorous account of the enrichment displayed by women for these genetic parameters can be found in the Supplementary material (Supplementary Fig. 4 and Supplementary Table 7a for heritability; Supplementary Fig. 5 and Supplementary Table 7b for evolvability).

As many brain IDBPs were found to be significantly phenotypically associated with MSCP in women, we hypothesised that the associations might be driven, at least partially, by genetic correlations between the associated traits. To test this, we estimated the genetic correlation between the 2319 IDBPs and MSCP in a sex-stratified manner (Supplementary Table 8a). Genetic correlations between IDBPs and MSCP were found slightly more negatively associated in women compared with men (*P*_KS_=10^−7^; Fig. 4e). Also, stronger genetic correlations, both positives and negatives, were found for women (Fig. 4e). These genetic correlations correlated somewhat poorly between men and women (28% variance explained; *P*<10^−16^; Fig. 4f). Thus, despite displaying correlated intersex genetic heritability estimates for IDBPs (Fig. 4b), men and women largely differ in their genetic correlations between IDBPs and chronic pain (Fig. 4f).

We then investigated whether strong phenotypic associations between IDBPs and MSCP could be partially explained by strong genetic correlations. For this purpose, we relied on a G×P plot (Fig. 4g). As genetic correlations can be positive or negative, as for phenotypic associations, the scatter plot now featured four quadrants (Q1–Q4). Visually, we observed that most points lay along the main diagonal, along the Q1–Q3 axis, indicating concordance of effect direction for both genetic and phenotypic associations. Women displayed many more IDBPs with simultaneous large genetic correlations and phenotypic associations than men (Fig. 4g). The partitioning of the scatter plot confirmed the finding, as women dominated counts of IDBPs in both the lower left (Q3) and upper right (Q1) corners of the plot (Fig. 4h). A more rigorous account of the enrichment put forth in women for genetic associations between IDBPs and MSCP can be found in the Supplementary material (Supplementary Table 8a; Supplementary Fig. 6). We found similar results of enrichment for women when considering shared causal variants between IDBPs and MSCP (Supplementary Fig. 7; Supplementary Table 8b), and when considering genetic causality of genetically correlated IDBPs with MSCP (Supplementary Fig. 8; Supplementary Tables 8c–e). Strikingly, in both men and women, estimates of genetic causality proportion implied that the genetics of brain features were causative for MSCP, and as such for most IDBPs (Supplementary Fig. 8a), with a causal enrichment observed in women for IDBPs strongly associated with MSCP (Supplementary Fig. 8c and d).

**Fig 5:**
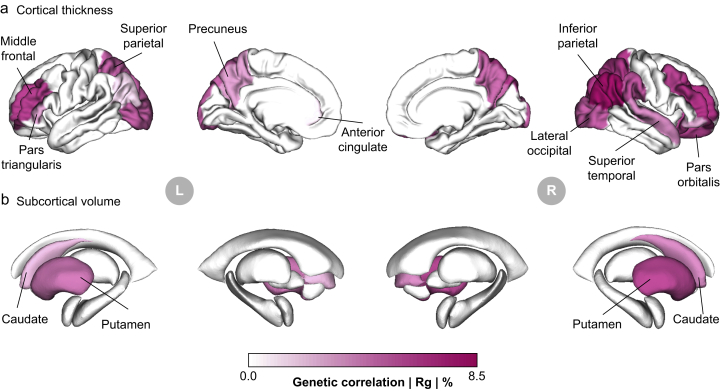
Brain maps of imaging-derived brain phenotypes (IDBPs) genetically correlated with multi-site chronic pain (MSCP). Cortical thickness (a) and subcortical volumes (b) are shown for women only as no significant associations were found in men. Colours track magnitudes of genetic correlations, with darker hues indicating larger correlations on the absolute scale (|R_g_|). Significant regions are annotated.

To fully explore the extent of the genetic couplings between IDBPs and MSCP, we used brain colourmaps. Specifically, cortical thickness phenotypes, identified as significantly enriched among the top 10% FDR-associated IDBPs with MSCP in women, were used to render cortical surface maps (Fig. 5a). In women, we found many brain regions whose cortical thicknesses were genetically correlated with MSCP, to up to the 8.5% level. In contrast in men, none of the IDBPs pertained to cortical thickness. A similar picture arose for measurements of subcortical volume, in which women again displayed some genetic correlations with MSCP among striatal substructures including the caudate nucleus and putamen (Fig. 5b). No subcortical structures were significantly genetically correlated with MSCP in men. These findings underscore that the genetically correlated brain alterations associated with chronic pain predominantly occur in women and align with commonly reported associations in areas such as the striatum,^62^ prefrontal cortex,^63^ and occipital lobe,^64^ highlighting a potential sex-specific pattern in the neural components of chronic pain.

Taken altogether, our results demonstrated that women featured more IDBPs phenotypically associated with MSCP with larger heritability estimates and stronger genetic correlations than men. The genetic architectures of IDBPs thus seemed to be intertwined with chronic pain, as demonstrated by the joint, but sex-specific distributions of genetic and phenotypic association strengths.

## Discussion

We used genetics to investigate why and how sex differences are observed in the human experience of chronic pain. So far, few genetics studies have tested pain traits in a sex-stratified manner.^14^^,^^15^ Nonetheless, our results suggest that sex-stratified genetic analyses provide novel insights elusive from sex-combined ones, and thus, sex-stratified analyses should be performed whenever possible. We found greater heritability in women most likely driven by higher polygenicity. Furthermore, we identified the most heritable pain trait as MSCP.

The sizable heritability of MSCP is likely because the phenotype is originated by body site-agnostic core biological processes that trigger and maintain chronic pain and are governed at the genetics level. These core processes are probably implicated in ‘pain spreading’,^7^ perhaps stemming from shared genetic etiologies,^33^^,^^65^^,^^66^ one of which was previously identified as the axonal guidance pathway.^6^ Interestingly, among the tested MSCP models, including previously published quantitative (QMCP^32^) and binary (MSCP^6^) ones, MSCP was found most heritable (Fig. 1c), alluding to a greater clinical and biological significance.

Sex differences in heritability have already been reported, with women displaying significantly higher heritability than men for several clinical phenotypes, including many blood pressure-related traits,^67^ and for regional volume and surface area phenotypes in the brain.^68^ However, neuropsychiatric and behavioural traits were generally found more heritable in men than in women but not significantly so, with a few exceptions in which women's heritability was significantly larger than men's, including recurrent major depressive disorder and post-traumatic stress disorder.^59^ Many psychiatric traits were found to be genetically correlated with chronic pain in a sex-specific manner, including post-traumatic stress disorder, autism, and schizophrenia.^14^ Genetic differences between sexes from the analyses of autosomal chromosomes could be puzzling at first sight but can be rationalised by sex-specific patterns of gene expression.^69^, ^70^, ^71^

The search for chronic pain-relevant tissue(s) led us to the brain, confirming the findings from previous genetic studies,^14^^,^^28^ but now demonstrating that these brain-specific signals are more prominent in women by far (Fig. 2b). Our results regarding the sex-stratified distribution of tissue contribution to pain states are of particular importance for future GWAS studies. They suggest that for women, contributing genetic signals to chronic pain are almost exclusively found in the brain, whereas for men, they are spread across multiple tissues, including brain ones. Although not significant in this study, the contribution of immune cells to pain states in men is very sizable and comparable with the nervous system, which warrants further research.

Comparing men and women at the level of IDBPs, we found that IDBPs were more strongly associated with MSCP in women (Fig. 3c). That phenomena appeared to be brain-wide, that is, all probed brain regions with different imaging modalities indicated stronger association with chronic pain in women than in men (Fig. 3d and e). Indeed, cortical thickness,^72^ orientation dispersion,^73^ and regional brain volumes^74^ were already reportedly associated with the presence of pain, but the sex-dependent nature of these associations was not explored.

The results above sparked a search for a deeper understanding of the relationship between the genetics of brain features and those of chronic pain. Indeed, Cheverud's conjecture^75^^,^^76^ provides a rationale for finding a genetic link between brain features and chronic pain, following the strong association of many IDBPs with the presence of chronic pain in women. Fortunately, it has been already shown that many brain features were genetically inherited,^29^^,^^30^ thus begging the question of whether the genetics of brain features correlated with the presence of chronic pain, and if so, were they sex-specific.

When genetic properties of IDBPs were explored, we found that IDBPs that were strongly phenotypically associated with MSCP were also heritable, evolvable, genetically correlated, shared more causative variants, and genetically causative, always in greater numbers in women than in men, with few exceptions (Fig. 4d and h; Supplementary Figs 4d, 5d, 6d, 6e, 7d, 8d, and 8e). As a general trend, we noted that the variability in IDBPs owing to genetics was often causative for chronic pain rather than the other way around (Supplementary Fig. 8a). Thus, genetics ‘wire’ the brain for chronic pain, especially in women (Supplementary Fig. 8c and d).

Phenotypic and genetic enrichment analyses underscored the importance of cortical integrity, including both thickness and area, as key factors involved in chronic pain. Sex-stratified genetic correlations revealed sex-differentiated neuroanatomical structures associated with chronic pain, with stronger associations in women, particularly in areas such as the precuneus, prefrontal cortex, anterior cingulate, and occipital lobe (Fig. 5a). The magnitudes of observed genetic correlation estimates between various IDBPs and MSCP up to levels of 8% were similar to those published between IDBPs and many neuropsychiatric traits and diseases, such as intelligence and neuroticism, bipolar disorder, and depressive symptoms.^77^ Extensive research has linked these regions to the processing and modulation of chronic pain, highlighting their roles in the emotional and cognitive aspects of pain perception.^78^^,^^79^ Furthermore, subcortical structures showed a clear bias towards women in terms of volumes associated with MSCP, with the striatum exhibiting a stark female bias in phenotypic association (Fig. 5b). Subcortical regions in the dorsal striatum including the putamen and caudate nucleus were highlighted because of their significant associations and roles in several dimensions of chronic pain. These regions are integral to the brain's pain processing network, with the putamen involved in reward and motor aspects of pain and pain processing, and the nucleus caudate involved in the cognitive modulation of pain.^80^^,^^81^ Their strong associations hint at a complex interplay between emotional, cognitive, and motor responses in chronic pain, deeper rooted in genetics for women than for men. These findings necessitate further research to unravel the underlying pathophysiological mechanisms related to structural changes in the brain across various chronic pain conditions within and between sexes.

The present study had multiple limitations. First, it cannot be surely determined if control subjects were truly pain-free (incomprehensive list of body sites at field 6159). Second, many factors were not accounted for, in particular past experiences of chronic pain, the type of pain (nociceptive *vs* nociplastic *vs* neuropathic), its origin and duration,^7^^,^^82^ sex-specific occupational hazards, employment types, developmental trajectories, and exposure to environmental factors such as pollutants.^83^, ^84^, ^85^, ^86^, ^87^, ^88^ Third, there is an order of magnitude smaller sample size of the cohort of brain study (imaging visit ∼35 000 individuals) compared with the baseline cohort (first assessment visit ∼350 000 individuals). Fourth, the focus on individuals of Caucasian ancestries leading to the concern about whether the conclusions drawn here also hold for other ancestries.^89^^,^^90^ Fifth, individuals were dichotomised by their sex chromosomes, either XX or XY; it has now become clear that gender and sex are not always concordant.^91^ Sixth, the participation biases in the UKB cohort and the brain imaging subcohort, in particular the one known as the ‘healthy volunteer’ bias, might push the prevalence estimates for chronic pain on the conservative side.^92^, ^93^, ^94^ Additionally, an internal selection bias has been noted in the follow-up imaging visit, with participants being sociodemographically comparable yet generally in better health than those from the initial baseline visit.^95^ Overall, these selection biases may distort association estimates, thus limiting the generality of the findings.^96^^,^^97^ Seventh, the inability of latent causal variable to infer true genetic causation direction and to deal with confounders was a limitation. Eighth, a replication study is needed. Ninth, the genetic contributions from the sex chromosomes were not assessed. On the one hand, chromosome Y would only apply to genetically determined men. On the other hand, chromosome X brings many analytical challenges and assumptions related to GWAS.^98^, ^99^, ^100^

Taken altogether, the large body of results presented here is a quantitative step towards explaining why and how women experience more chronic pain than men: genetic factors increase the risk for chronic pain in women significantly more than in men.

## Authors’ contributions

Study conceptualisation: MP, MR, EVP, LD

Bioinformatics analyses: MP

Data visualisation: MP, MF

Interpretation of results: MP, MF, CTS, MR, EVP, LD

Manuscript drafting: MP, MF, CTS, LD

Funding acquisition: MR, EVP, LD

Read, revised, and approved the final version of the manuscript: all authors

## Funding

Funding provided by: a professorship in Pain Research from Pfizer Canada to LD; a Canadian Excellence Research Chairs grant (CERC09) to LD; an NIH grant (U54 DA049110) to LD; a Canada Research Chair (tier 2) on Brain Imaging of Experimental and Chronic Pain to MR; and a CIHR grant (453096) to EVP.

## Declaration of interest

LD is a consultant for Duke University, ONO PHARMA USA Inc., Releviate Inc., and Orthogen AG. All other authors declare that they have no competing interests.
