# Supplementary material for: Sex-specific genetics underlie increased chronic pain risk in women: genome-wide association studies from the UK Biobank
Source: Br J Anaesth. 2025 May 22;135(2):401–15. doi: 10.1016/j.bja.2025.04.013 (PMC12308093; doi:10.1016/j.bja.2025.04.013)
Supplement: Multimedia component 1 [file mmc1.pdf]

Sex-Specific Brain Genetics Underlie Increased Chronic Pain Risk in Women

Marc Parisien, Matthew Fillingim, Christophe Tanguay-Sabourin, Mathieu Roy, Etienne Vachon-Presseau, Luda Diatchenko

Supplementary Materials

SUPPLEMENTARY METHODS

Evolvability

Adaptation to evolutionary pressure can be estimated using the evolutionary response to selection.[1] It is distinct from and uncorrelated with heritability.[2] Evolvability is defined as $e=\sigma_g^2/\mu_p^2$, where σ_g^2 is the additive genetic variance and μ_p the trait's phenotypic mean. We swapped σ_g^2 with the following, knowing that the narrow-sense heritability h^2 is defined as: $h^2= \sigma_g^2/\sigma_p^2$, the ratio of genetic to phenotypic variances (the variance is the squared standard deviation). Thus $e=h^2 \cdot \sigma_p^2/\mu_p^2=h^2 \cdot (\sigma_p/\mu_p)^2$, with σ_p standing for the standard deviation of the trait. The ratio σ/μ is the inverse of what's called the signal-to-noise ratio (μ/σ).

Genetic Causality Proportion

A further step in the quantification of shared genetic architecture is asking if one phenotype is genetically causative of the other phenotype, or if they simply share a pleiotropic genetic effect. At the genome-wide level, LCV (latent causal variable) was devised to answer such a question and estimate what's termed the genetic causality proportion (GCP). The GCP is a signed value between -1 and +1, distinguishing causality (minus or plus one) from pleiotropy (zero) between two genetically correlated traits.[3] We emphasized "genome-wide" since there exist other tools to detect genetic causality, but at the individual variants level (e.g. CAUSE [4]). For the purposes of our study, we estimated the GCP between imaged-derived phenotypes and the presence of multi-site chronic pain. A positive value of the GCP indicated that the IDBP was not only genetically correlated with but was also causative for MSCP (noted as IDBP ⇒ MSCP), and a negative value indicated that MSCP was causative for the IDBP (MSCP ⇒ IDBP). Values near zero were indicative of genetic pleiotropy (rather than causation) in the face of significant genetic correlation. Quality controls used to boost the credibility of genetic causality proportions were, as described in the LCV paper,[3] minimum absolute Z-score of 1.96 (P-value of 0.05) for total genetic correlation between the IDBP and MSCP (traits have to be nominally genetically correlated), the absolute value of total genetic correlation of no more than 0.9 (traits have to be correlated but not too much), FDR-corrected P-value for narrow-sense heritability of IDBP less than 10% (trait 1 has to be heritable), and Z-score for narrow-sense heritability of MSCP greater than 7 (trait 2 has to be heritable; heritability Z-score for MSCP is about 25).

Genotype-by-Phenotype Plot

To study the relationship between heritability and phenotypic association we relied on a 'Genotype-by-Phenotype' (GxP) plot. The plot is a scatter plot, where the X axis represents the IDBP's test statistic for phenotypic association with MSCP, and the Y axis represents IDBP's heritability estimate (or any other genetic parameter of interest). For phenotypic associations, sample sizes for men and women were matched, and so test statistics were employed for X coordinates. For genetic estimates, since women's sample size was slightly larger than men's (as we retained all participants to preserve statistical power), we relied on the estimate itself rather than its associated test statistic for Y coordinates. Inflated test statistics in favour of women by a

factor of $\sqrt{16{,}580} / \sqrt{14{,}770} = 1.06$, or 6% were expected simply due to the greater number of women (all other factors being equal, as $Z=\beta/SE$, but $SE=SD/\sqrt{N}$, so $Z \propto \sqrt{N}$). We employ curves to track constant values of kappa (κ), defined as the product of X and Y coordinates: $\kappa = X \cdot Y$. The 1-dimensional κ value tells how much an IDBP is simultaneously genetically and phenotypically related to chronic pain. From the plot, we're then allowed to ask how many data points have a kappa value greater or equal to a value of interest. At large kappa values in the GxP plots, we visually appreciated the presence of more data points for women compared to men. To quantify this phenomenon, we tracked the number of IDBPs in both women (N_W) and men (N_M) along all values of kappa, asking how many IDBPs have $X \cdot Y \geq \kappa$, with the log2 value of the N_W/N_M ratio defining the enrichment.

SUPPLEMENTARY RESULTS

Sex differences in the genetic architectures of IDBPs

We performed sex-stratified GWAS on 2319 IDBPs in the UK Biobank cohort. From the summary GWAS results we extracted the narrow-sense heritability estimates (Supplementary Fig. 4A; Supplementary Table 7A). Sex-stratified distributions of heritability estimates were found to not significantly differ overall ($P_{KS}>0.05$), but women featured more IDBPs with larger heritability estimates than men in the 30%-45% heritability range. Nonetheless, heritability estimates in women correlated with those in men, with a percent variance explained of 56% ($P<10^{-16}$) (Supplementary Fig. 4B).

Genetic features of an IDBP become relevant to our study only when it is strongly phenotypically associated with chronic pain. We developed a Genotype-by-Phenotype (GxP) plot to visualize and quantify the relationship between IDBPs and chronic pain. The single most important genetic feature of a trait is its genetic heritability. We thus tracked the genetic heritability of an IDBP versus its phenotypic association with chronic pain in a GxP plot (Supplementary Fig. 4C). There, we qualitatively observed more IDBPs in women that are simultaneously heritable and phenotypically associated with chronic pain. To quantify this enrichment, we tracked the number N of IDBPs whose product of X and Y coordinates were greater than a fixed value, kappa (κ; curved gold lines), that in a sex-stratified fashion (N_W and N_M, in women and men respectively) (Supplementary Fig. 4D). We found that women consistently featured more IDBPs, for kappa values greater than about 0.3, within both quadrants Q1 and Q2. For $\kappa \geq 0.6$, the enrichment in women is at least 4-fold greater.

To complement heritability analyses, we also investigated sex differences in the evolvability of IDBPs. The evolvability of a trait can be loosely defined as how much it is allowed to drift (think standard deviation). Like the case of heritability, we also found no significant differences in the sex-stratified distributions of evolvability ($P_{KS}>0.05$) (Supplementary Fig. 5A; Supplementary Table 7B). Evolvability in men and women largely correlated (82% variance explained, $P<10^{-16}$) (Supplementary Fig. 5B). Interestingly, IDBPs that corresponded to structural connectivity were those that featured the largest values of evolvability, in both men and women (Supplementary Fig. 5B). However, the GxP plot indicated that women possessed many IDBPs simultaneously strongly associated with MSCP and that were highly evolvable (Supplementary Fig. 5C). Indeed, for $\kappa \geq 13$,

women displayed an enrichment in number of IDBPs compared with men, that to a level of 2-fold more (Supplementary Fig. 5D).

Sex differences in the genetic co-architectures between IDBPs and MSCP

We estimated the genetic correlation between the 2319 IDBPs and MSCP in a sex-stratified fashion (Supplementary Table 8A). Genetic correlations between IDBPs and MSCP were found slightly more negatively associated in women compared to men ($P_{KS}=10^{-7}$; Supplementary Fig. 6A). Also, stronger genetic correlations, both positives and negatives, were found for women (Supplementary Fig. 6A). The genetic correlations were somewhat poor between men and women (28% variance explained; $P<10^{-16}$; Supplementary Fig. 6B).

We next looked at how genetically correlated IDBPs to MSCP were also phenotypically associated (Supplementary Fig. 6C). Since genetic correlations can be positive or negative, as for phenotypic associations, the GxP plot now featured four quadrants (Q1 to Q4). Visually, we observed that most points lay along the main diagonal, along the Q1-Q3 axis. Women displayed many more IDBPs with simultaneous large genetic correlations and phenotypic associations than men. That was quantified for the main diagonal comprising quadrants Q1 and Q3 (Supplementary Fig. 6D), where women had four times more data points from $\kappa\geq1.5$. Women also dominated in Q4 (Supplementary Fig. 6E). Interestingly, data points for men were more abundant in Q2 (Supplementary Fig. 6E).

To complement genetic correlation analyses, we considered the estimated shared number of causal variants, between each IDBPs and MSCP, in a sex-stratified fashion. Like the case of heritability, we also found no significant differences in the sex-stratified distributions of shared causal variants ($P_{KS}>0.05$) (Supplementary Fig. 7A; Supplementary Table 8B). The number of shared causal variants in men and women moderately correlated (27% variance explained, $P<10^{-16}$) (Supplementary Fig. 7B). The GxP plot indicated that women possessed many IDBPs simultaneously strongly associated with MSCP and shared lots of causal variants (Supplementary Fig. 7C). Indeed, for $\kappa\geq2$, women displayed enrichment in number of IDBPs compared with men, and that to a level of 2-fold more for positively associated pairs in Q1 for all values of κ (Supplementary Fig. 7D).

After establishing the genetic correlations between chronic pain and brain features, we aimed to determine if one of these traits is genetically causative of the other or if they simply share a pleiotropic genetic effect. We utilized the latent causal variable (LCV) algorithm to estimate the genetic causality proportion (GCP) between MSCP and each one of the 2319 IDBPs in a sex-stratified fashion (Supplementary Table 8C). For both men and women, the distributions of GCP heavily leaned on the positive side, implying that the genetics of brain features were causative for MSCP, and such for most IDBPs (Supplementary Fig. 8A). Also, when MSCP was deemed causative for IDBPs, the GCP values barely reached -0.5, far from the unequivocal -1.0. Although the mean GCP in men was +18.9% whereas +19.0% in women, the overall sex-specific curves significantly differed ($P_{KS}=7\times10^{-6}$). We haven't found any significant sex differences on a per-IDBP basis that reached the FDR 10% level (Supplementary Table 8C). However, as a trend, men

largely dominated the MSCP ⇒ IDBP regime as well as some moderate values in the IDBP ⇒ MSCP regime.

In women, 36 IDBPs were deemed significant at the FDR 10% level (Supplementary Table 8D). Of those, all of them also featured positive GCPs suggesting that changes in IDBP lead to MSCP, and 33 of those (92%) had negative genetic correlation coefficients, indicating that these IDBPs were mostly anti-correlated to MSCP. In men, 37 IDBPs were found significant at the FDR 10% level (Supplementary Table 8E). Of those, all of them featured positive GCP values implying that the IDBPs were causative for MSCP. 25 of the 37 (68%) displayed negative genetic correlation coefficients.

Genetic causality proportions were entirely uncorrelated between men and women (percent variance explained 0.5%) (Supplementary Fig. 8B). The proportions were then reinterpreted in the context of phenotypic associations using a GxP plot (Supplementary Fig. 8C). As expected from the densities of GCP values, quadrants Q1 and Q2 displayed many more data points than quadrants Q3 and Q4. The scatter plot then helped to establish that when the IDBP was causative for MSCP (GCP > 0; Q1 and Q2), women displayed enrichment of IDBPs compared to men for $\kappa \geq 0.75$, especially in Q1 where the enrichment was at least 2-fold for most kappa values (Supplementary Fig. 8D). However, counts of IDBPs in Q3 were more prominent in men (Supplementary Fig. 8E). Overall, high kappa values were enriched in women in three of the four quadrants, indicating that genetic causation beyond correlation is in effect between IDBPs and MSCP pairs of traits, more often in women than in men.

SUPPLEMENTARY LEGENDS

Supplementary Figure 1 Genome-wide association study genomic inflation factors. Chronic pain conditions are: Presence of Site with Chronic Pain (PSCP, blue), Single-Site Chronic Pain (SSCP, yellow), Multi-Site Chronic Pain (MSCP, green), Adjacent-Site Chronic Pain (ASCP, reddish purple), and Quantitative Multisite Chronic Pain (QMCP, orange). Sex strata are: men and women combined (M+W), women-only (W), and men-only (M). Shown are the linkage-disequilibrium-uncorrected genomic inflation factors or λ_{GC} (upward-pointing triangles), the linkage-disequilibrium-corrected genomic inflation factors or LD score regression's intercepts (downward-pointing triangles), and confounding bias ratios (circles). All results from LDSC.

Supplementary Figure 2 Sex differences in the genetic architecture of chronic pain. Chronic pain phenotypes are: Presence of Site with Chronic Pain (PSCP, blue), Single-Site Chronic Pain (SSCP, yellow), Multi-Site Chronic Pain (MSCP, green), Adjacent-Site Chronic Pain (ASCP, reddish purple), and Quantitative Multisite Chronic Pain (QMCP, orange). Sex strata are: men and women combined (M+W), women-only (W), and men-only (M). * when P-value for difference is $< 5\times10^{-2}$, non-significant (ns) otherwise. **(A)** Narrow-sense heritability estimates and their standard errors. Results from LDSC. **(B)** Number of estimated causal variants and their standard errors. Results from univariate MiXeR. **(C)** The number of causal variants unique to each sex and common to both sexes for MSCP. Results from bivariate MiXeR. Also reported are the genetic (Rg) and environmental (Re) correlation coefficients (in percentages). Results from GECKO. **(D)**

Discoverability estimates and their standard errors. Results from univariate MiXeR. **(E)** Polygenicity and their standard errors. Results from univariate MiXeR.

Supplementary Figure 3 Sex differences in the tissue-based partitioned heritability of chronic pain. (left) Average heritability <h^2> per SNP at loci of genes specifically expressed in selected tissues. The 106 tissues or cell lines from Benita et al. are grouped by: central nervous system (C, purple, n=21), peripheral nervous system (P, blue, n=4), muscle (M, red, n=3), stem cells (S, green, n=11), myeloid cells (Y, orange, n=16), B cells (B, brown, n=8), T cells (T, pink, n=22), and other tissues or cell lines (O, dark grey, n=21). Insignificant tissues with FDR > 10% are coloured light grey. The top plot is for women, bottom plot is for men. (right) Relative contributions of tissue classes to partitioned heritability, in women (left) and in men (right). For simplicity, only the central nervous system (C, purple) slices were annotated in the pies. * when P-value < 0.05 for the difference in heritability between men and women for the central nervous system class, not significant (ns) otherwise. Chronic pain models are: **(A)** Presence of Site with Chronic Pain (PSCP); **(B)** Single-Site Chronic Pain (SSCP); **(C)** Multi-Site Chronic Pain (MSCP); **(D)** Adjacent-Site Chronic Pain (ASCP); and **(E)** Quantitative Multisite Chronic Pain (QMCP).

Supplementary Figure 4 Sex differences in genetic heritability of IDBPs. (A) Sex-stratified distributions of heritability estimates of IDBPs. **(B)** Correlation of heritability estimates between men (X-axis) and women (Y-axis). **(C)** Sex-stratified GxP plots. A few selected kappa (κ) values are shown as gold curves. **(D)** Enrichment plots. Shown are the log2 ratio of counts of IDBPs in women (N_W) to counts in men (N_M) as a function of κ, for each GxP plot quadrant separately.

Supplementary Figure 5 Sex differences in genetic evolvability of IDBPs. (A) Sex-stratified distributions of evolvability estimates of IDBPs. **(B)** Correlation of evolvability estimates between men (X-axis) and women (Y-axis). **(C)** Sex-stratified GxP plots. **(D)** Enrichment plots.

Supplementary Figure 6 Sex differences in the genetic correlations between IDBPs and MSCP. (A) Sex-stratified distributions of genetic correlation coefficients between IDBPs and MSCP. **(B)** Correlation of genetic correlation coefficients between men (X-axis) and women (Y-axis). **(C)** Sex-stratified GxP plots. **(D, E)** Enrichment plots.

Supplementary Figure 7 Sex differences in the shared causal variants between IDBPs and MSCP. (A) Sex-stratified distributions of the number of shared causal variants between IDBPs and MSCP. **(B)** Correlation of shared number of causal variants between men (X-axis) and women (Y-axis). **(C)** Sex-stratified GxP plots. **(D)** Enrichment plots.

Supplementary Figure 8 Sex differences in the genetic causality proportions between IDBPs and MSCP. (A) Sex-stratified distributions of genetic causality proportions (GCP) between IDBPs and MSCP. **(B)** Correlation of GCPs between men (X-axis) and women (Y-axis). **(C)** Sex-stratified GxP plots tracking GCP as a function of phenotypic association between IDBPs and MSCP. **(D)** Enrichment plots for which IDBP is causative for MSCP (IDBP ⇒ MSCP). **(E)** Enrichment plots for which MSCP is causative for IDBP (MSCP ⇒ IDBP).

Supplementary Table 1 Genome-wide association study genomic inflation factors. Columns are: ‘pain’, the chronic pain phenotype; ‘sex’, the sex stratification; ‘LDSC_ratio’, the ratio of;

‘LDSC_intercept’, the intercept of the regression of the GWAS’ chi^2 on LD scores or the LD-corrected genomic inflation factor; ‘lambda_GC’, the genomic inflation factor uncorrected for LD.

Supplementary Table 2 Body site-agnostic models for chronic pain. (A) Surveyed chronic pain sites and generalized chronic pain models. Columns are: ‘UKB’, the UK Biobank field code; ‘sex’, the sex stratum; ‘nb_cases’, the number of case subjects (for binary traits); ‘nb_ctrls’, the number of control subjects (for binary traits); ‘nb_total’, the total number of subjects in the GWAS (for both binary and quantitative traits); ‘trait’, the trait’s type; and ‘body_site’, the location of the chronic pain site on the human body. **(B)** Overrepresentation of women in chronic pain. The overrepresentation is estimated from a logistic regression between sex and chronic pain, corrected for age, body-mass index and index of multiple deprivation. Columns are: ‘UKB’, the UK Biobank field code; ‘body_site’, the location of the chronic pain site on the human body; ‘beta’, the effect size of the chronic pain term; ‘se’, its standard error; ‘Z’, its test statistic; ‘P’, its raw P-value; ‘FDR’, the false discovery rate-corrected P-value; ‘OR’, the odds ratio; ‘OR_lo’, the 95% confidence interval lower limit on the OR’s point estimate; ‘OR_hi’, the 95% confidence interval upper limit. **(C)** Heritability of chronic pain at each body site and for the generalized pain models. Columns are: ‘UKB’, the UK Biobank field code; ‘h2_pe_M’, the narrow-sense heritability point estimate in men; ‘h2_se_M’, its standard error; ‘h2_P_M’, its raw P-value; ‘h2_FDR_M’, its FDR-corrected P-value; ‘h2_pe_W’; the narrow-sense heritability point estimate in women; ‘h2_se_W’, its standard error; ‘h2_P_W’, its raw P-value; ‘h2_FDR_W’, its FDR-corrected P-value; ‘dZ’, the test statistic of the sex difference in heritability; ‘dP’, its raw P-value; ‘dFDR’, its FDR-corrected P-value; ‘bias’, the log2 ratio of heritability in women to men; a positive value indicates larger heritability in women compared to in men; ‘prev_M’, the observed prevalence of cases in men; ‘h2_lia_M’, the heritability estimate on the liability scale assuming so ascertainment (population prevalence = sample prevalence); ‘prev_W’, the observed prevalence of cases in women; ‘h2_lia_W’, the heritability estimate on the liability scale. QMCP is a quantitative trait, so conversion from observed- to liability-scaled heritability estimates doesn’t apply.

Supplementary Table 3 The genetic architecture of chronic pain. (A) Narrow-sense heritability estimates. Columns are: ‘pain’, the chronic pain phenotype; ‘sex’, the sex stratification; ‘h2_pe’, the heritability’s point estimate; ‘h2_se’, the heritability’s standard error; ‘h2_P’, the heritability’s P-value; ‘diff_P’, the P-value for the sex difference in heritability estimates. **(B)** MiXeR univariate results for narrow-sense heritability. Columns are: ‘pain’, the chronic pain phenotype; ‘sex’, the sex stratification; ‘h2 (mean)’, the mean of 20 replicates for the point estimate of narrow-sense heritability due to causal SNPs; ‘h2 (se)’, the estimate’s standard error; ‘nc@p9 (mean)’, the mean for the point estimate of the number of causal SNPs explaining 90% of the heritability; ‘nc@p9 (se)’, the estimate’s standard error; ‘diff_P_nc@p9’, the P-value for the sex difference in the number of causal variants; ‘sig2_beta (mean)’, the mean for the point estimate of discoverability; ‘sig2_beta (se)’, the estimate’s standard error; ‘diff_P_sig2_beta’, the P-value for the sex difference in discoverability; ‘pi (mean)’, the mean for the point estimate of polygenicity; ‘pi (se)’, the estimate’s standard error; ‘diff_P_pi’, the P-value for the sex difference in polygenicity. **(C)** MiXeR bivariate results for narrow-sense heritability. Columns are: ‘stratum’, the stratum (pain or sex) in which the analysis was performed; ‘versus’, the two contrasted strata; ‘nc1@p9 (mean)’, the mean of 20 replicates for the point estimate of the number of causal SNPs explaining 90% of the heritability unique to the left stratum; ‘nc1@p9 (se)’, the estimate’s standard error; ‘nc2@p9 (mean)’, the mean for the point estimate of the number of causal SNPs explaining 90% of the

heritability unique to the right stratum; ‘nc2@p9 (se)’, the estimate’s standard error; ‘diff_P’, the P-value for the difference (of sex or chronic pain model) between the number of unique causal variants nc1 and nc2; ‘nc12@p9 (mean)’, the mean for the point estimate of the number of causal SNPs explaining 90% of the heritability shared between the two strata; ‘nc12@p9 (se)’, the estimate’s standard error; ‘rg (mean)’, the mean for the point estimate of the global genetic correlation at shared causal SNPs; ‘rg (se)’, the estimate’s standard error. **(D)** Genetic and environmental correlations estimated with GECKO. Columns are: ‘stratum’, the stratum (pain or sex) in which the analysis was performed; ‘versus’, the two contrasted strata; ‘Env_PE’, the environmental correlation point estimate; ‘Env_SE’, the estimate’s standard error; ‘Env_P’, the estimate’s P-value; ‘Gen_SE’, the genetic correlation point estimate; ‘Gen_SE’, the estimate’s standard error; ‘Gen_P’, the estimate’s P-value.

Supplementary Table 4 Tissue-based partitioned heritability of chronic pain. (A-J) Partitioning heritability generated on the Benita et al. tissues dataset. Columns are: ‘Name’, the name of the tissue or cell type; ‘Class’, the broader class assignment of the tissue or cell type; ‘Coefficient’, the tau_C value or average heritability at SNPs in genes specifically expressed in the cell type or tissue; ‘Coefficient_std_error’, the coefficient’s standard error; ‘Coefficient_P_value’, the coefficient’s raw P-value; ‘FDR’, the coefficient’s FDR-corrected P-value. **(A)** PSCP in women. **(B)** PSCP in men. **(C)** SSCP in women. **(D)** SSCP in men. **(E)** MSCP in women. **(F)** MSCP in men. **(G)** ASCP in women. **(H)** ASCP in men. **(I)** QMCP in women. **(J)** QMCP in men. **(K-O)** Sex differences in heritability enrichment by tissues. Columns are: ‘name’, the name of the tissue or cell type; ‘class’, the class of tissues; ‘coef_W’, the tau_C value or average heritability at SNPs in genes specifically expressed in the cell type or tissue in women; ‘coef_W_SE’, the coefficient’s standard error; ‘coef_M’, men’s enrichment coefficient; ‘coef_M_SE’, the coefficient’s standard error; ‘diff_Z’, the standard error-weighted sex difference of enrichment coefficients; ‘diff_P’, the associated P-value; ‘diff_FDR’, the FDR-corrected P-value. **(K)** Tissue-based sex difference for PSCP. **(L)** Tissue-based sex difference for SSCP. **(M)** Tissue-based sex difference for MSCP. **(N)** Tissue-based sex difference for ASCP. **(O)** Tissue-based sex difference for QMCP. **(P-T)** Sex differences in heritability enrichment by classes of tissues. Columns are: ‘Class’, the class of tissues; ‘Sum.M’, the sum of partitioned heritability coefficients across all tissues of the same class in men; ‘SE.M’, the sum’s standard error; ‘pct.M’, the sum’s percentage; ‘Sum.F’, the sum of partitioned heritability coefficients across all tissues of the same class in women; ‘SE.F’, the sum’s standard error; ‘pct.F’, the sum’s percentage; ‘dZ’, the Z-score for the difference in coefficients between men and women; ‘dP’, the difference’s P-value; ‘dFDR’, the FDR-adjusted P-values. **(P)** Class-based sex difference for PSCP. **(Q)** Class-based sex difference for SSCP. **(R)** Class-based sex difference for MSCP. **(S)** Class-based sex difference for ASCP. **(T)** Class-based sex difference for QMCP.

Supplementary Table 5 Phenotypic association between IDBPs and MSCP. (A) Logistic regression between normalized (Z-scored) IDBPs and MSCP was performed, one IDBP at a time. Columns are: ‘beta_W’, the logistic regression effect size in women; ‘se_W’, the associated standard error; ‘pval_W’, the associated P-value; ‘padj_W’, the FDR-corrected P-value; ‘beta_M’, the logistic regression effect size in men; ‘se_M’, the associated standard error; ‘pval_M’ the associated P-value; ‘padj_M’, the FDR-corrected P-value; ‘stat_D’, the test statistic of the difference in the effect sizes between women and men; ‘pval_D’, the associated P-value; ‘padj_D’, the FDR-corrected P-value. Each row is an IDBP.

Supplementary Table 6 Sex bias in the association strengths between IDBPs and MSCP. (A-D) Results of enrichment of IDBP labels via hypergeometric test. Columns are: 'label', the label tested for enrichment; 'q', the number of IDBPs whose description featured the label and is also FDR 10% significant; 'm', the total number of IDBPs whose description featured the label; 'n', the total number of IDBPs whose description not featured the label; 'k', the number of IDBPs that were FDR 10% significant; 'nrch', the enrichment value of the label, with values larger than one indicating an over-representation; 'pval', the P-value of the enrichment, obtained using the hypergeometric test; 'padj', the FDR-adjusted P-value. **(A)** Enrichment results for labels for brain modalities in women, for the 197 IDBPs that were FDR ≤ 10% significant. **(B)** Enrichment results for labels for brain modalities in men, for the 6 IDBPs that were FDR ≤ 10% significant. **(C)** Enrichment results for labels for brain regions in women, for the 75 IDBPs that were FDR ≤ 10% significant and were attributed to a brain region. **(D)** Enrichment results for labels for brain regions in men, for the 2 IDBPs that were FDR ≤ 10% significant and were attributed to a brain region. **(E-F)** Analysis of sex bias. Columns are: 'label', the label tested for bias; 'N', the number of IDBPs whose description featured the label; 'Δ(W-M)', the measured bias between women's and men's phenotypic association with chronic pain test statistic data. **(E)** Sex bias in the modalities of IDBPs. **(F)** Sex bias in brain regions. **(G)** Brain regions of origin for the various IDBPs. Adapted from Table S2 of Lin et al.[5]

Supplementary Table 7 The genetic architecture of IDBPs. (A) Narrow-sense heritability estimates (h^2). Columns are: 'fname', the code name of the IDBP in UKB; 'h2..mean..M', the mean for the point estimate for the heritability in men; 'h2..se..M', the estimate's standard error; 'h2..mean..W', the mean for the point estimate for the heritability in women; 'h2..se..W', the estimate's standard error; 'h2..dlog2', the log2 ratio of heritability estimates in men compared with women; 'h2..dZ', the test statistic of the difference between sex-stratified estimates; 'h2..dP', the associated P-value; 'h2..dFDR', the FDR-corrected P-value; 'code', the UKB code of the IDBP; 'desc', the IDBP's short description. **(B)** Evolvability estimates. Columns are: 'fname', the code name of the IDBP in UKB; 'n2s_M', the squared noise-to-signal ratio $(sd/m)^2$ of the IDBP, with sd the standard deviation of the trait and m the trait's mean, in men; 'h2_M', the narrow-sense heritability estimate of the IDBP in men; 'e_M', the evolvability estimate of the IDBP in men; 'n2s_W', the squared noise-to-signal ratio $(sd/m)^2$, in women; 'h2_W', the narrow-sense heritability estimate in women; 'e_M', the evolvability estimate in women. Statistical assessment of the sex differences has not been attempted since the standard errors of evolvability estimates have not been calculated.

Supplementary Table 8 Genetic co-architecture between IDBPs and MSCP. (A) Genetic correlation between MSCP and brain IDBPs. The genetic correlations between MSCP and IDBPs are presented in a sex-stratified manner. The coloured sections are: blue for men; purple for women; and yellow for the sex difference in genetic correlations. Columns are: 'brain', the brain IDBP; 'pain', the correlated pain phenotype; 'gen_PE.M', the genetic correlation point estimate in men (from GECKO); 'gen_SE.M', the estimate's standard error; 'gen_P.M', the estimate's P-value; 'gen_PE.F', the genetic correlation point estimate in women (from GECKO); 'gen_SE.F', the estimate's standard error; 'gen_P.F', the estimate's P-value; 'gen..dZ', the Z-score of the difference in the genetic point estimates between men and women; 'gen..dP', the associated P-value; 'gen_dFDR', the FDR-corrected P-value. **(B)** Results of MiXeR's bivariate analyses

indicating the estimated number of shared causal variants between MSCP and the IDBPs. Columns are: ‘brain’, the ID of the IDBP; ‘nc12_W’, the estimated number of shared causal variants in women; ‘nc12_M’, the estimated number of shared causal variants in men. **(C-E)** Genetic causality proportions between MSCP and brain IDBPs. Causality estimated between MSCP and IDBPs, in a sex-stratified manner. **(C)** Differences in genetic causality proportions between men and women on a per-IDBP basis. Columns are: ‘serial’, the UKB-assigned serial number of the IDBP; ‘gcp.pm.M’, the genetic causality proportion estimate in men (from LCV); ‘gcp.pse.M’, the estimate’s standard error; ‘gcp.pm.F’, the genetic causality proportion in women (from LCV); ‘gcp.pse.F’, the estimate’s standard error; ‘gcp.pm.dZ’, the Z-score of the difference between men and women; ‘gcp.pm.dP’, the associated P-value; ‘gcp.pm.dFDR’, the FDR-corrected P-value; ‘desc’, a short description of the brain IDBP. **(D-E)** Genetic causality proportions estimated by LCV. Columns are: ‘serial’, the UKB-assigned serial number of the IDBP; ‘gcp.pm’, the genetic causality proportion estimate, when positive means that the brain IDBP is causal for MSCP; ‘gcp.pse’, the estimate’s standard error; ‘gcp.pval’, the estimate’s P-value; ‘gcp.padj’, the FDR-corrected P-value; ‘rho.est’, the global genetic correlation coefficient estimate between the IDBP and MSCP; ‘rho.err’, the estimate’s standard error; ‘h2.zscore.trait1’, the Z-score of the narrow-sense heritability estimate from LCV for the brain IDBP; ‘h2.zscore.trait2’, same as previous but for MSCP; ‘QC’, indicates if the genetic causality proportion should be trusted based on quality controls; ‘h2.trait1’, estimate for the IDBP’s narrow sense heritability from LDSC; ‘h2_se.trait1’, the estimate’s standard error; ‘h2_pval.trait1’, the estimate’s P-value; ‘h2_padj.trait1’, the FDR-corrected P-value; ‘desc’, a short description of the brain IDBP. **(D)** Genetic causality proportions in women. **(E)** Genetic causality proportions in men.

SUPPLEMENTARY REFERENCES

1 Garcia-Gonzalez, F., Simmons, L. W., Tomkins, J. L., Kotiaho, J. S. & Evans, J. P. Comparing evolvabilities: common errors surrounding the calculation and use of coefficients of additive genetic variation. *Evolution* **66**, 2341-2349 (2012). https://doi.org:10.1111/j.1558-5646.2011.01565.x

2 Hansen, T. F., Pélabon, C. & Houle, D. Heritability is not Evolvability. *Evolutionary Biology* **38**, 258-277 (2011). https://doi.org:10.1007/s11692-011-9127-6

3 O'Connor, L. J. & Price, A. L. Distinguishing genetic correlation from causation across 52 diseases and complex traits. *Nat Genet* **50**, 1728-1734 (2018). https://doi.org:10.1038/s41588-018-0255-0

4 Morrison, J., Knoblauch, N., Marcus, J. H., Stephens, M. & He, X. Mendelian randomization accounting for correlated and uncorrelated pleiotropic effects using genome-wide summary statistics. *Nat Genet* **52**, 740-747 (2020). https://doi.org:10.1038/s41588-020-0631-4

5 Lin, S. *et al.* Inferring the genetic relationship between brain imaging-derived phenotypes and risk of complex diseases by Mendelian randomization and genome-wide colocalization. *Neuroimage* **279**, 120325 (2023). https://doi.org:10.1016/j.neuroimage.2023.120325

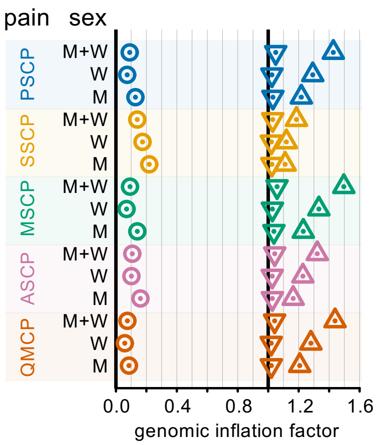

Supplementary Figure 1

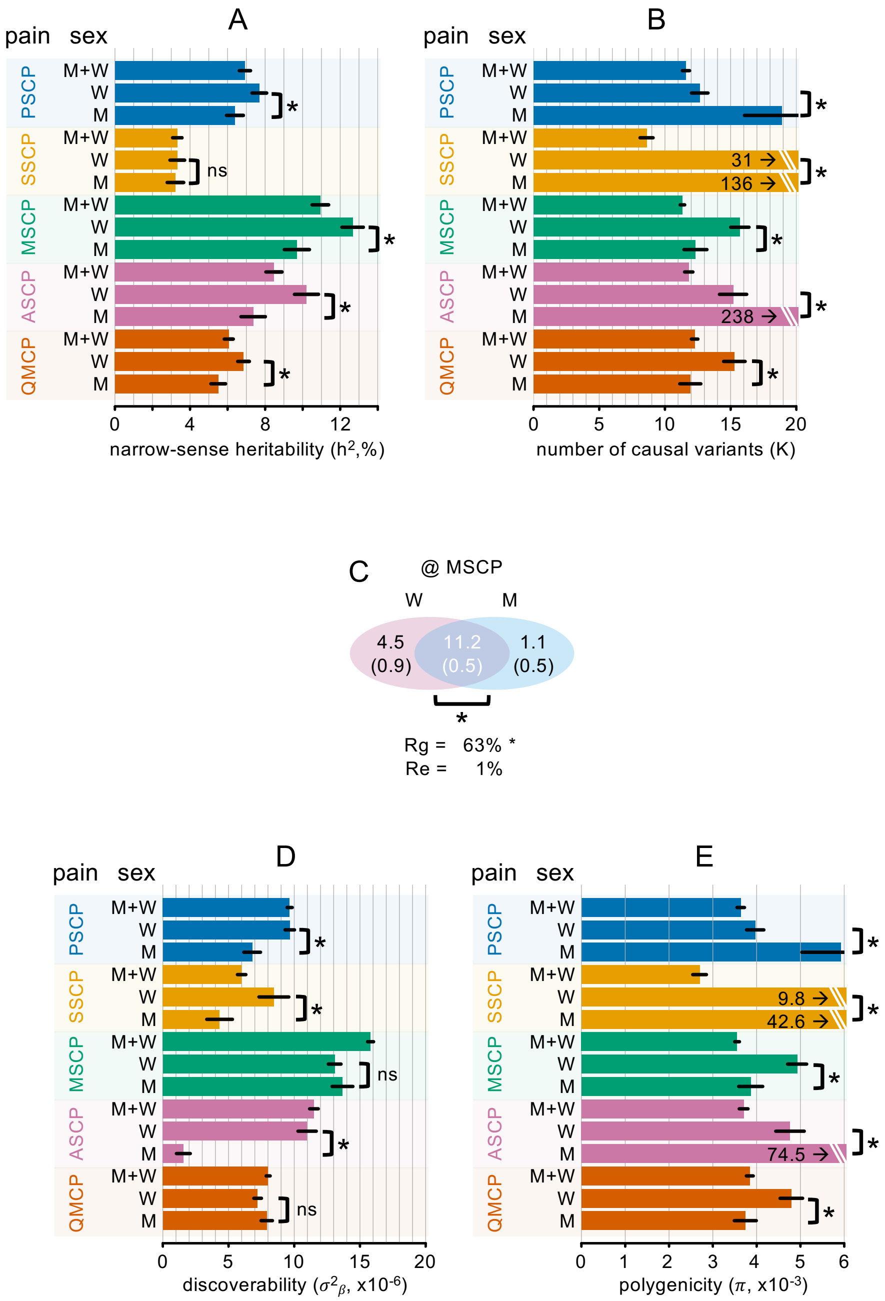

Supplementary Figure 2

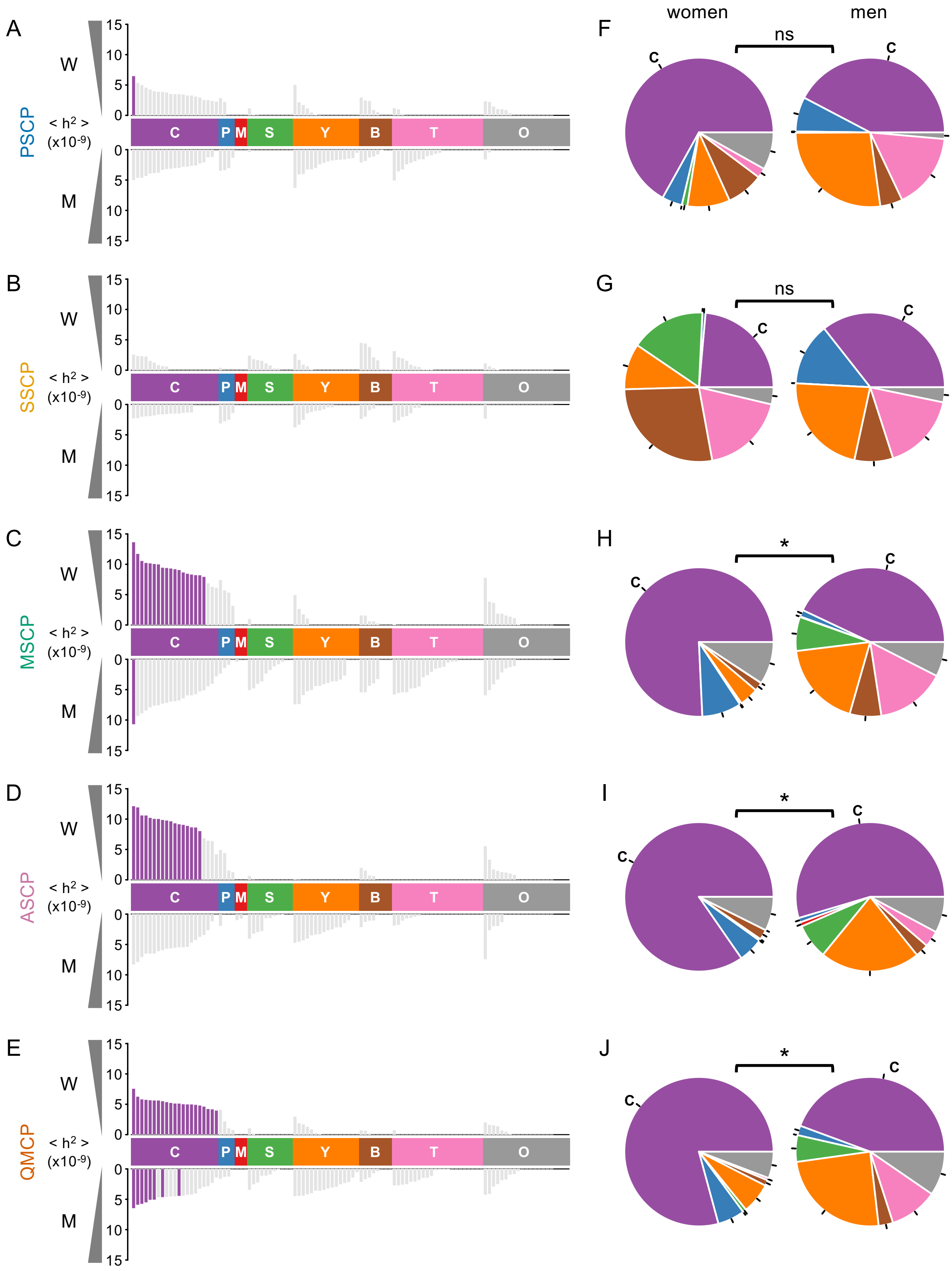

Supplementary Figure 3

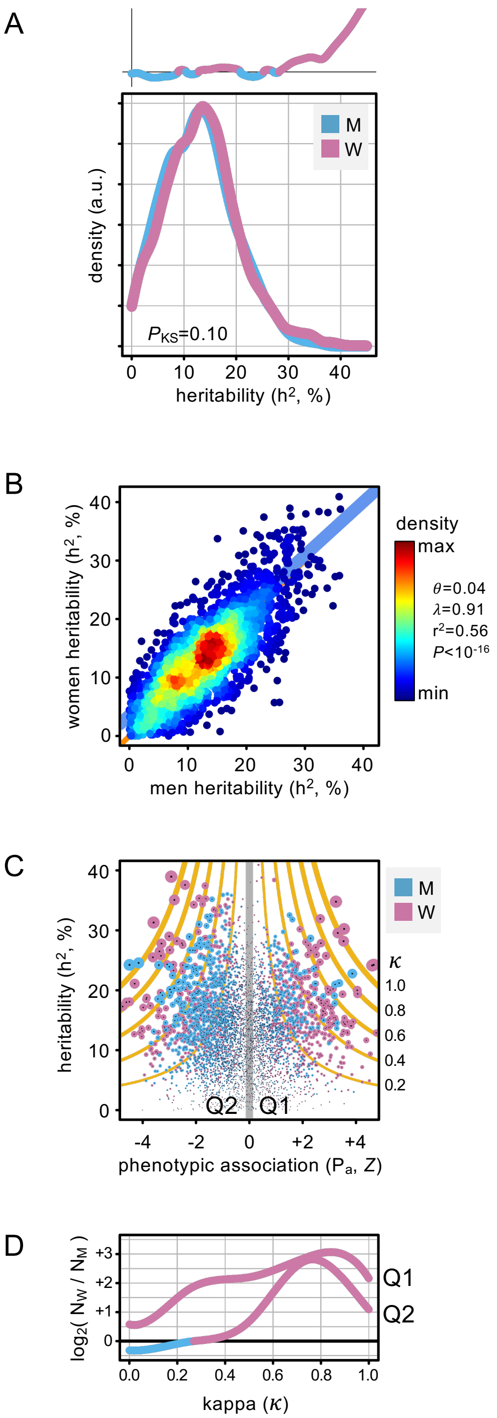

Supplementary Figure 4

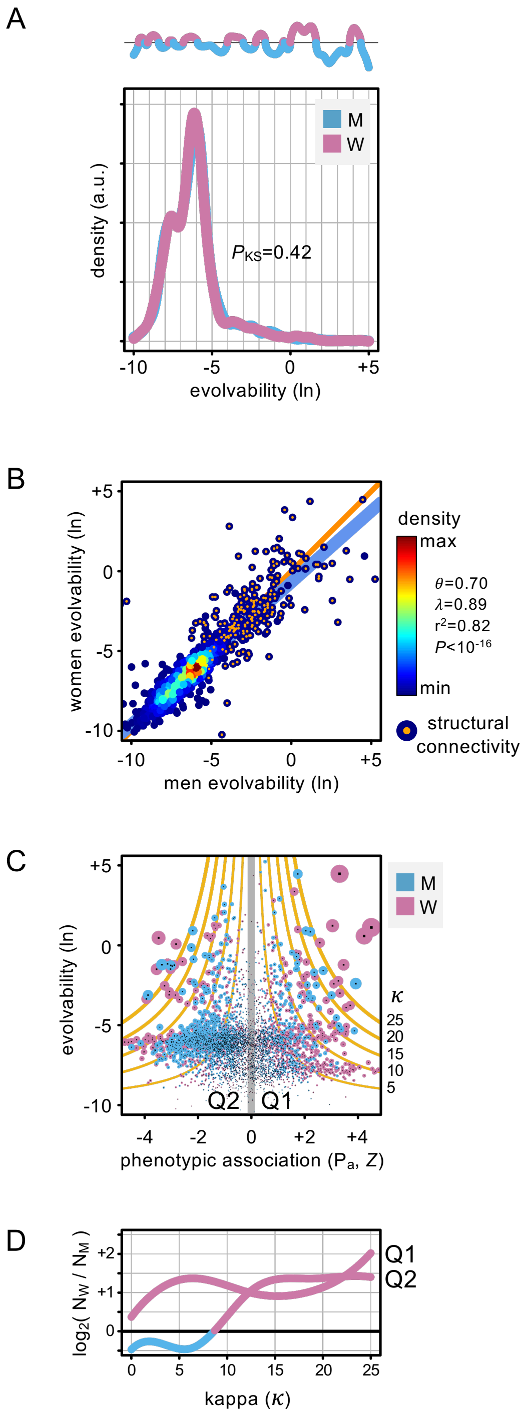

Supplementary Figure 5

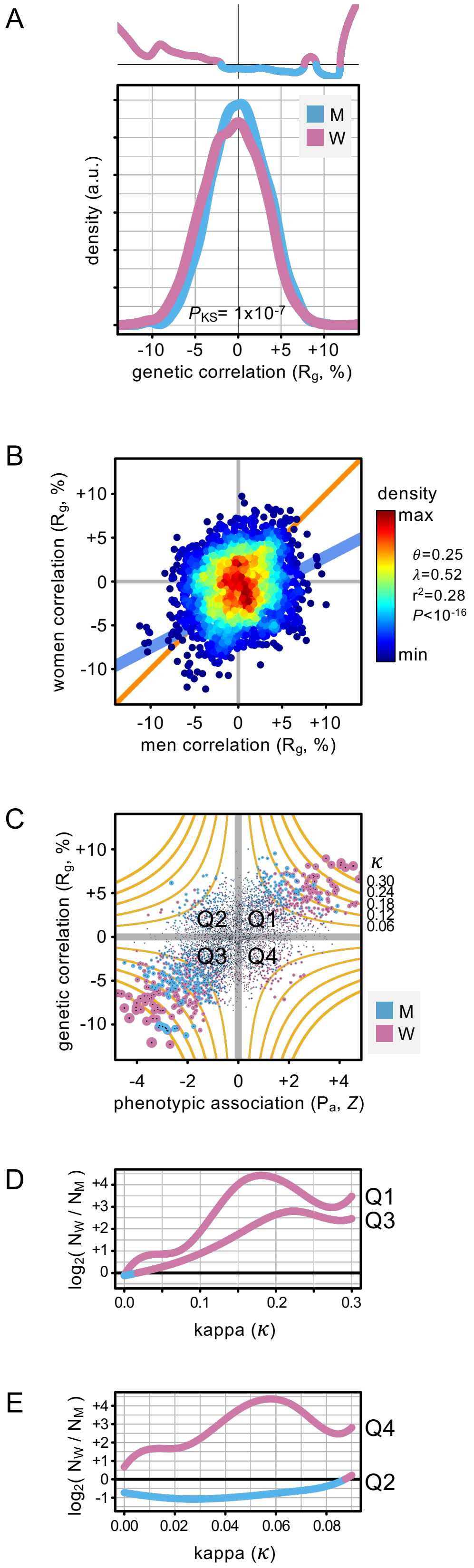

Supplementary Figure 6

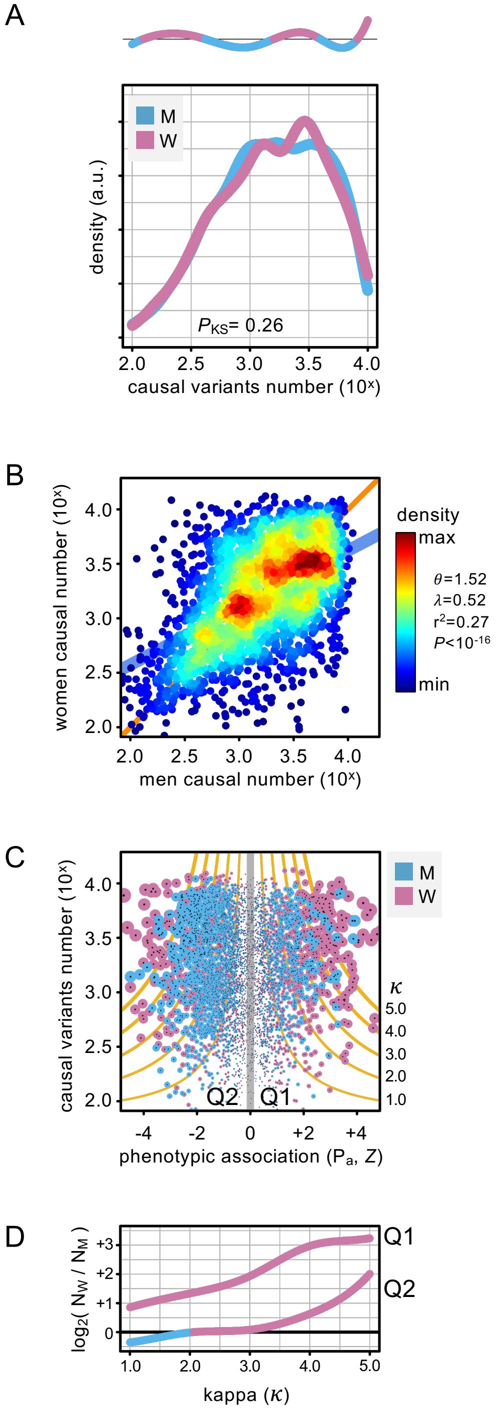

Supplementary Figure 7

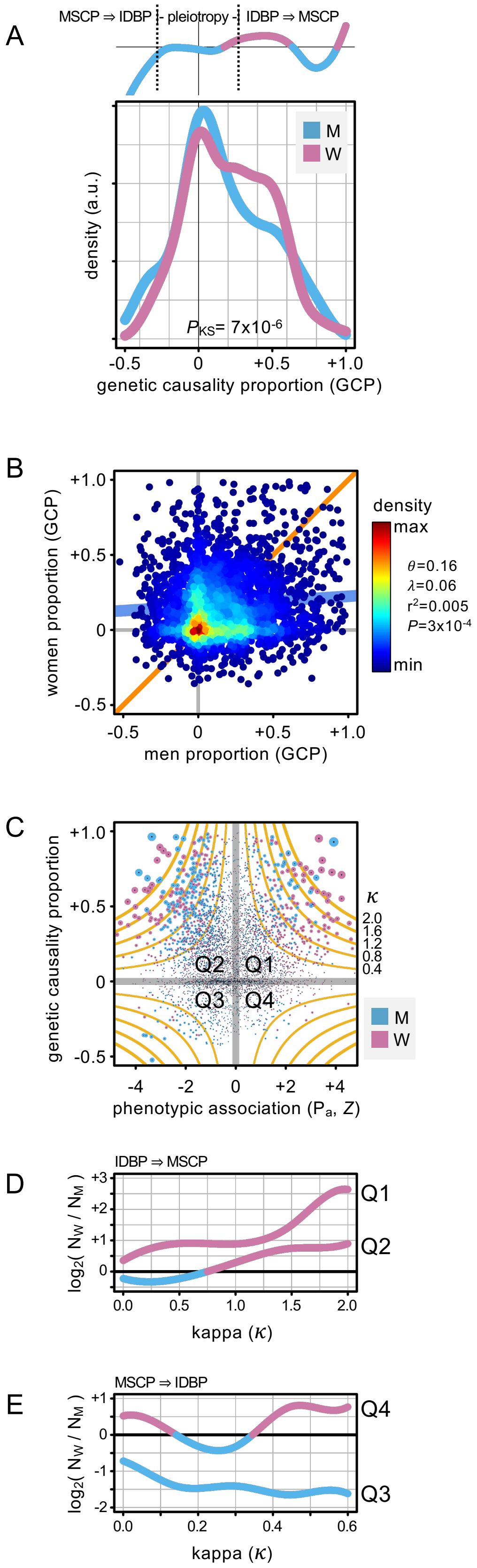

Supplementary Figure 8
